# Conbase: a software for unsupervised discovery of clonal somatic mutations in single cells through read phasing

**DOI:** 10.1186/s13059-019-1673-8

**Published:** 2019-04-01

**Authors:** Joanna Hård, Ezeddin Al Hakim, Marie Kindblom, Åsa K. Björklund, Bengt Sennblad, Ilke Demirci, Marta Paterlini, Pedro Reu, Erik Borgström, Patrik L. Ståhl, Jakob Michaelsson, Jeff E. Mold, Jonas Frisén

**Affiliations:** 1 Department of Cell and Molecular Biology, Karolinska Institutet, Solna, Sweden; 2 Department of Cell and Molecular Biology, National Bioinformatics Infrastructure Sweden, Scilifelab, Uppsala University, Uppsala, Sweden; 3 Division of Gene Technology, Scilifelab, KTH Royal Institute of Technology, Solna, Sweden; 4 Center for Infectious Medicine, Department of Medicine, Karolinska Institutet, Huddinge, Sweden

**Keywords:** Single-cell DNA sequencing, Single-cell variant calling, Somatic variation

## Abstract

Accurate variant calling and genotyping represent major limiting factors for downstream applications of single-cell genomics. Here, we report Conbase for the identification of somatic mutations in single-cell DNA sequencing data. Conbase leverages phased read data from multiple samples in a dataset to achieve increased confidence in somatic variant calls and genotype predictions. Comparing the performance of Conbase to three other methods, we find that Conbase performs best in terms of false discovery rate and specificity and provides superior robustness on simulated data, in vitro expanded fibroblasts and clonal lymphocyte populations isolated directly from a healthy human donor.

**Electronic supplementary material:**

The online version of this article (10.1186/s13059-019-1673-8) contains supplementary material, which is available to authorized users.

## Background

Single-cell DNA sequencing has substantial untapped potential for understanding genomic diversity in both health and disease [1, 2]. A particularly interesting target application for single-cell genomics is lineage tracing of human cells, where the distributions of somatic variants among cells in a population reflect their phylogenetic relationships. In this context, minimizing the number of falsely predicted somatic variants is of utmost importance as these introduce noise or misleading signals, severely reducing the power of phylogenetic analyses. Current methods for single-cell DNA sequencing typically require a whole genome amplification (WGA) step to yield enough DNA for sequencing. WGA is widely recognized to introduce technical artifacts including allelic dropout, amplification bias and amplification errors leading to false negative and false positive genotype predictions [3–6]. However, while WGA may introduce amplification errors, which in turn may result in false positive variant calls, amplification errors will most likely affect only one cell because of the low likelihood that exactly the same error arises in more than one WGA reaction. Error rates in WGA have been estimated to correspond to one error per 10^5^–10^6^ bases in multiple displacement amplification (MDA) and three errors per 10^4^ bases in multiple annealing and looping-based amplification cycles (MALBAC) [7]. A more abundant source of false positive variant calls in whole genome sequencing data results from failed realignment and the limitation of using a reference genome with respect to the genome of the donor, giving rise to 1 error in 10–15 kb in raw variant calling output [2, 4, 8–10]. The typical effect of these alignment artifacts is that a locus may be covered by reads originating from multiple locations in the genome, for instance due to paralogous sequences, structural variants that are not present in the reference genome, or a location in low-complexity regions [2, 4, 8–10]. While it is recommended to discard variant calls present in regions in which alignment artifacts occur [2, 4, 8–10], it can be extremely difficult to detect their presence just based on the reads of a given cell. Somatic mutations in normal, non-malignant, cells are generally estimated to be infrequent, corresponding to approximately three somatic mutations per cell division [2]. Thus, the expected number of false positive variant calls far exceeds the predicted number of true somatic mutations. Moreover, expected observations in WGA data are sites covered by reads originating from only one of the two alleles, due to allelic dropout [3–6]. Sites displaying reads originating from only one allele may result in false negatives if dropout occurs only for the mutated allele. Taken together, alignment artifacts, amplification errors, and allelic dropout result in false positive variant calls and incorrect genotype predictions, hampering the use of variant calling to define phylogenetic relationships at the single-cell level [4, 5].

In order to address artifacts in whole genome sequencing data from related cells, we developed a computational strategy for the unsupervised discovery of somatic single nucleotide variants (sSNVs) in single cells. Accurate genotyping of the individual single cells is achieved independently of the global rate of allelic dropout. Conbase is a multistep algorithm that confirms the allelic origin of bases through read phasing, by using the abundant signal from germline single-nucleotide variants (gSNVs) across the genome. The discovery of sSNV sites is based on analysis of observed haplotype concordance within individual single cells, across the population of cells and in an unamplified bulk sample. By further exploiting the phasing information, locus-specific allelic dropout is determined per sample individually, enabling exclusion of false negative genotypes resulting from dropout of the mutated allele. Thus, Conbase is an ideal tool for detecting mutations in closely related cells from the same individual. However, it is not designed for the analysis of unrelated cells that do not share any somatic mutations.

We evaluate the performance of Conbase on simulated data and two different real datasets containing single-cell DNA libraries from healthy human cells with known phylogenetic relationships prepared using different WGA techniques. For comparative reasons, we evaluate the performance of three additional methods for variant calling in single cells, including Monovar [4], SCcaller [5], and Linked Read Analysis (LiRA) [11]. Monovar leverages data across a single-cell dataset to detect sSNV sites and predicts the presence or absence of mutations in individual samples. This is done by modeling the effects of errors arising from WGA and assuming that data from different loci are independent. SCcaller models amplification bias using read depth observations in gSNV sites to estimate likelihoods that a variant call is an artifact or a sSNV. SCcaller calls variants independently per sample and does not predict the absence of mutations in unmutated samples. Like Conbase, LiRA utilizes read phasing to correct for errors and allelic dropout. In contrast to Conbase and Monovar, and in accordance with SCcaller, LiRA does not perform joint variant calling across the population of cells. While read phasing, as used by Conbase and LiRA, can enable lower false discovery rate (FDR) and higher specificity, it comes with the cost of only enabling analysis of bases in proximity to gSNVs, whereas SCcaller and Monovar has the potential to detect sSNVs in all bases covered by reads. It is worth noting that LiRA and SCcaller only predicts the presence of variants in cells, while absence of a variant (the ancestral, unmutated state) is conflated with missing prediction. In contrast, Conbase and Monovar predict both mutated and unmutated states of cells. Information about the absence of somatic mutations in unmutated cells in a dataset is required for many downstream applications for single-cell genomics, including phylogenetic analysis and high-resolution lineage tracing at the single-cell level.

In summary, we demonstrate the effectiveness of Conbase for identifying true sSNVs in single-cell DNA sequencing libraries exhibiting varying degrees of allelic dropout, generated from biologically relevant populations of human cells. We believe that Conbase will be an increasingly valuable tool for applications ranging from phylogenetic analysis of single eukaryotic cells on the basis of acquired sSNVs to the characterization of the mutational landscape of single cells in healthy and diseased tissues.

## Results

### Overview of Conbase variant calling

**Fig. 1:**
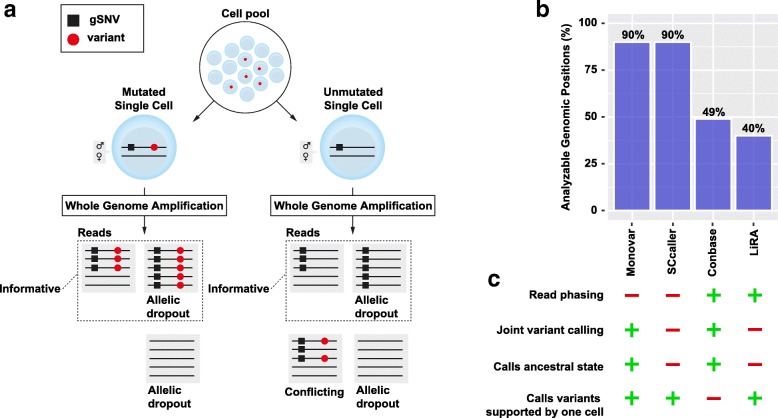
**a** Somatic mutations are present in a subset of a population of cells and can be identified by DNA sequencing of whole genome amplified single cells. WGA may result in allelic dropout, which in turn may result in false negative variant calls if dropout has occurred of the mutated allele. False positive variant calls may arise from amplification errors or alignment artifacts among molecules with high sequence similarity, resulting in conflicting haplotype observations. Conbase circumvents these problems by determining locus-specific allelic dropout individually per sample and analyzes concordance of the observed haplotypes across the cell population. **b** The percentage of genomic positions that are analyzable by Conbase, Monovar, SCcaller, and LiRA. Genomic positions analyzable for Monovar and SCcaller were defined as the fraction of bases covered by at least one read in an unamplified bulk sample sequenced at 40x coverage. Genomic positions analyzable for Conbase and LiRA were defined as the fraction of unique genomic positions present within 650 bp of gSNVs. **c** Overview of some features of Conbase, Monovar, SCcaller, and LiRA

Allelic dropout, amplification errors, and alignment artifacts can result in false negative and false positive genotype calls, respectively (illustrated in Fig. 1). Conbase aims to circumvent these problems by integrating phasing and analysis of observed haplotype concordance during variant calling (Fig. 1, Additional file 1 Figure S1). Phasing putative sSNVs to gSNVs allows for the determination of maternal or paternal origin of variants, because true sSNVs are expected to be observed only on either the maternal or the paternal allele in the population of cells. The allele that harbors a variant in mutated samples is here defined as the informative allele. The informative allele is distinguished from the non-informative allele by the base observation in gSNVs present in the same sequenced molecule (Fig. 1, Additional file 1 Figure S1). The genotypes of samples that only display reads originating from the non-informative allele are unknown (Fig. 1, Additional file 1 Figure S1). False negative variant calls, resulting from allelic dropout, are eliminated by requiring that a sample have reads originating from the informative allele in order to be assigned a genotype (Fig. 1, Additional file 1 Figure S1). False positive variant calls result from amplification errors and systematic errors, including alignment artifacts. These are to a large extent excluded by analyzing multiple aspects of observed data during variant calling and genotyping, including monitoring observed haplotypes in putative sSNV loci (Fig. 1, Additional file 1 Figure S1), maximal expected read depth in an unamplified bulk sample, and the density of mismatches against the reference genome in the region in which variant calls are present (“Methods” section: “Algorithm description” and “gSNV filtering”). The fraction of the genome that can be phased by gSNVs depend on the frequency and distribution of gSNVs in the genome of the donor, as well as on the average insert size of the single-cell sequencing libraries (Additional file 1 Figure S2). With an average sequencing library of 650 bp, ~ 50% of the genome can be phased by Conbase (Fig. 1b, Additional file 1 Figure S2). LiRA only takes gSNVs that are present in dbSNP into account; hence, the phasable proportion for LiRA is slightly lower (Fig. 1b). Since Monovar and SCcaller are not dependent on read phasing, these methods have the advantage of enabling detection of sSNVs in all bases covered by reads (Fig. 1b, c). Like Monovar, Conbase utilizes data from multiple samples in a dataset to perform joint variant calling, and both these methods predict the absence of mutations in unmutated samples, in contrast to SCcaller and LiRA that only predicts the mutated state and does not distinguish missing data from unmutated states (Fig. 1c). In contrast to the other three methods, Conbase only calls clonal somatic variants (Fig. 1c); hence, it requires the presence of a mutation in at least two cells. Different features of the methods included in our comparative analyses are summarized in Fig. 1c.

### Performance evaluation on simulated data

We first evaluated the performance of the tested methods using simulated datasets. The advantage of using simulated data generated from a statistical model is that we control the important parameters, which allows us to properly estimate relevant statistics. However, a problematic issue is how to design a generative model that reasonably well reflects biological reality. Here, we simulated data from a model allowing us to control the proportion of two main causes for errors when calling clonal somatic variants in single-cell whole genome sequencing data: errors from alignment artifacts (EAL) and allelic dropout. To address the problem of biological reality, we based our simulation model on loci, reads, and coverage distributions in bulk and single-cell data obtained from a CD8+ T cell donor and a primary human fibroblast donor, later used in our analyses of experimental data. For each set of simulation experiments, we performed two variants with respect to coverage distribution. In our main variant, we used the empirical coverage distributions observed in single cells, to simulate data exhibiting amplification bias. To also investigate the impact of an even coverage distribution, our second variant used a flat coverage of 30 reads per site, representing optimal data quality with uniform amplification on both alleles in all loci. In the simulations, we focused on sSNV sites with phased data available. As noted above, methods based on phasing, including Conbase and LiRA, will fail to predict sSNVs that are not present in the same read or read pair as at least one gSNV, whereas Monovar and SCcaller has the potential of calling variants in all genomic positions covered by reads (Fig. 1b). This means that the sensitivity for variant calling, as defined below, would be lower if unphased data were included, while it would likely be unaffected for Monovar and SCcaller. Moreover, it is reasonable to assume that the specificity and FDR for all methods would not deviate substantially from those estimated on the phased data.

We performed two separate experiments testing different prediction targets, first focusing on the detection of clonal mutations across a population of cells and secondly on correct genotyping in each individual site. In our first experiment, we investigated the effect of EAL in combination with allelic dropout, on the correct and incorrect classification of loci as clonal sSNVs in a population of cells. We therefore simulated a population of cells consisting of two clones with 10 cells each and a set of loci (*n* = 306) comprising sSNV sites as well as an increasing fraction of sites that were homozygous for the reference allele and affected by EAL. In the sSNV sites, the true genotype for all cells of a randomly selected clone was heterozygous, representing a clonal sSNV, and all cells in the other clone were homozygous for the reference allele, representing the ancestral, unmutated state. No homozygous sSNVs were included in the simulations testing the accuracy of variant calling, because of the low likelihood that the same mutation occurs on both alleles in a diploid genome. The sites that were homozygous for the reference allele represent positions harboring the ancestral, unmutated state.

**Fig. 2:**
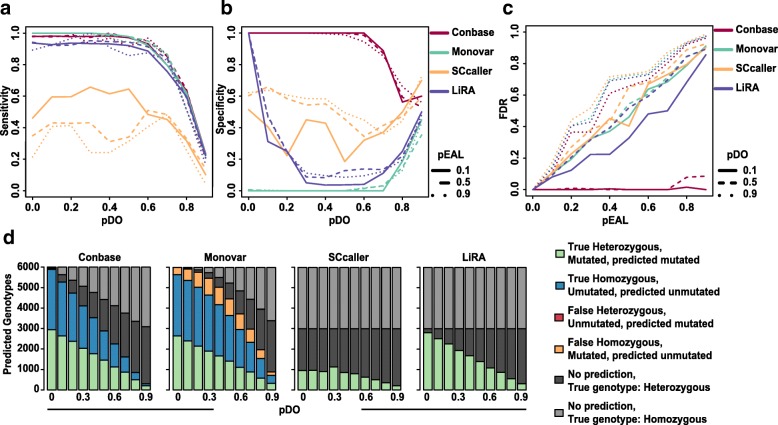
**a**–**c** The accuracy of variant calling was evaluated for Conbase, Monovar, SCcaller, and LiRA, reflecting the ability of the methods to detect clonal sSNV loci a population of cells. The sensitivity (**a**) and specificity (**b**) of Conbase, Monovar, SCcaller, and LiRA to detect clonal mutations in at least two cells in simulated data at increasing dropout probabilities (pDO) at different levels of alignment artifact probabilities (pEAL). **c** The false discovery rate of Conbase, Monovar, SCcaller, and LiRA when detecting clonal mutations in simulated data at increasing alignment artifact probabilities (pEAL) at different levels of dropout probabilities (pDO). **d** The accuracy of genotyping was evaluated for Conbase, Monovar, SCcaller, and LiRA, reflecting the ability of the methods to correctly predict genotypes in each sSNV loci and each cell at increasing dropout probabilities (pDO). In each simulation, representing one bar, the true genotype in 50% of the samples were heterozygous, representing samples harboring a sSNV. The remaining samples were homozygous for the reference allele, representing the ancestral unmutated state

Variant calling was performed on the simulated read data using Conbase, Monovar, LiRA, and SCcaller to obtain classification of the loci as clonal sSNVs, defined as sites in which at least two cells shared a variant. Sensitivity, specificity, and FDR was computed for each method under varying degree of EAL and dropout. The results show that Conbase, Monovar, and LiRA performs comparably in terms of sensitivity to detect sSNV sites, while the sensitivity of SCcaller is lower (Fig. 2a, Additional file 1 Figure S3a). When evaluating the accuracy of variant calling in data exhibiting even read depth coverage, we found that the sensitivity of SCcaller was substantially improved, indicating that data quality affects the sensitivity of SCcaller (Additional file 1 Figure S3b). The sensitivity, determined for SCcaller and Monovar in this simulation, correspond well with findings presented in another study comparing these methods [5]. Conbase outperforms Monovar, SCcaller, and LiRA in terms of the specificity to distinguish artifacts, except when the dropout probability is close to 1, in which case all methods approach a specificity of 0.5 (Fig. 2b, Additional file 1 Figure S3c). At low dropout probability, LiRA enables detection and exclusion of artifacts resulting from EAL, but its specificity then deteriorates rapidly with increasing dropout probability (Fig. 2b, Additional file 1 Figure S3c). For all tested methods, except for Conbase, FDR increases linearly with EAL. Conbase consistently achieves very low FDR except at extremely high dropout probability (Fig. 2c, Additional file 1 Figure S3d).

In the second experiment, we focused on the accuracy of the tested methods to correctly predict genotypes in individual cells in sSNV sites. We therefore simulated only sSNV loci without EAL, but with varying degree of allelic dropout, and generated reads from these. Here, we also simulated a population of cells consisting of two clones with 10 cells each with mutations occurring in one of the clones in the same manner as described above, but focused on extracting genotype prediction statistics from each individual site in each cell. Again, no homozygous sSNVs were included in the simulations, due to the low likelihood that the same mutation occurs on both alleles in a diploid genome. Conbase, Monovar, SCcaller, and LiRA were applied to the simulated data, and we recorded their classification of loci in individual cells as heterozygote (presence of a sSNV), homozygote (ancestral, unmutated state), or no prediction. We next evaluated the number of correctly and incorrectly predicted genotypes per site (Fig. 2d). In Conbase, Monovar, and LiRA output, we observe similar distributions of correctly predicted heterozygous genotypes, whereas SCcaller predicts a lower number of true heterozygotes. Notice that SCcaller and LiRA only predicts the presence of mutations, and therefore, no predictions are observed in the samples with simulated homozygous genotypes (Fig. 2d). Monovar detects a higher number of true homozygotes, as compared to Conbase (Fig. 2d). However, Monovar also predicts a higher number of false homozygotes as compared to Conbase (Fig. 2d). The false homozygotes predicted by Monovar are to a large extent mutated samples with dropout of the mutated allele. This scenario is distinguished from true homozygotes by Conbase, which makes no predictions in samples exhibiting dropout of the mutated allele. Also here, we performed a second variant of the simulation using even read depth coverage, in which all four methods perform better, in particular SCcaller (Additional file 1 Figure S3e).

### Performance evaluation on real data obtained from in vitro expanded human fibroblasts

To evaluate the performance on real data, we first made time-lapse recordings of primary human fibroblasts as they divided on polymer slides allowing us to subsequently identify and isolate single fibroblasts with known phylogenetic relationships by laser capture microscopy. We isolated 11 cells derived from clone 1, three cells derived from clone 2, and two unrelated cells. All these single cells were whole genome amplified by MALBAC [12]. The single-cell libraries were sequenced to obtain an average of 385 million reads per single cell, corresponding to approximately 12x coverage of the human genome on unamplified genomic DNA (Additional file 2 Table S1). We first estimated amplification efficiency relative to allelic dropout and locus dropout, by analyzing the fraction of genomic bases covered by reads and the fraction of gSNV sites covered by reads originating from the maternal allele, the paternal allele or from both alleles (Additional file 1 Figure S4a–c). On average, 26% of genomic bases were covered by at least one read, with a 70% allelic dropout rate at the covered gSNV sites (Additional file 1 Figure S4b, c). Although varying degrees of allelic dropout has been reported for MALBAC data [6, 12], the rates of allelic dropout observed in this experiment is likely to reflect the fact that the cells were harvested by laser capture microscopy, and thus, part of the genomic material may be lost in the isolation process.

We next performed variant calling on bulk genomic DNA and single fibroblast libraries using FreeBayes [13] and computed the fraction of sites in which an alternative genotype was observed in single-cell libraries but not in the bulk sample (Additional file 1 Figure S4d). These sites include a combination of true sSNVs and false positive variant calls. The unrealistically high number of variants uniquely called in single cells (551,971–1,220,408 unique variant calls per single cells) is suggestive of the presence of a large number of false positive variant calls. This is expected as MALBAC is reported to have a relatively high error rate due to the lack of proofreading by the *Bst* polymerase in the initial amplification steps, coupled with exponential amplification in the final steps of the protocol [12]. Moreover, variant callers designed for bulk data, including FreeBayes, do not account for the unique properties of WGA-amplified single-cell data and may result in inaccurate SNV calling [4, 5].

**Fig. 3:**
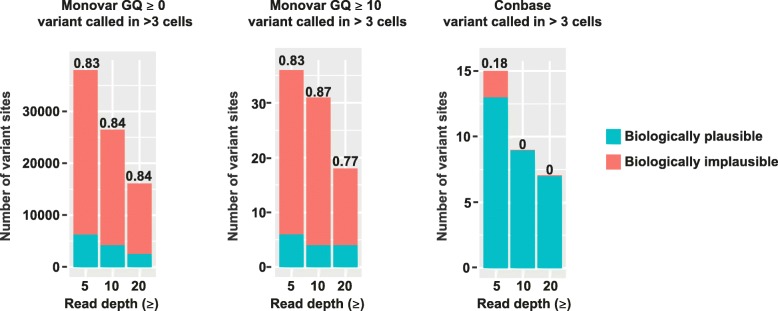
Biologically plausible and implausible distributions of genotypes called by Monovar and Conbase in clonal populations of fibroblasts. Values above bars represent false discovery rates. Biologically plausible genotype distributions were defined as sites where the variant call is exclusively observed within cells belonging to the same clone. Biologically implausible genotype distributions were defined as sites where the variant call is observed within both clones and at least one cell displayed the reference genotype

We next performed variant calling with Monovar and Conbase, which are designed to account for the errors and biases in WGA single-cell data. To estimate the FDR of these methods, we computed the fraction of sites in which the distribution of genotypes was biologically implausible in our clonal populations of fibroblasts. True sSNVs are expected to be shared by closely related clonal cells and not distributed between cells of different clones. Under the assumption that the probability of two mutations occurring independently in the same site twice is extremely low [14], we defined implausible genotype distributions as sites where a variant call was observed in both clones and at least one cell displayed the reference genotype. Variants that are restricted to a single clonal population represent a biologically plausible genotype distribution. Variants observed in both clones, without observing individual cells harboring the reference genotype, may however be gSNVs incorrectly interpreted as sSNVs due to the absence of variant supporting reads in the bulk sample since bulk sequencing data may also suffer from allelic dropout due to insufficient sequencing coverage. However, requiring that at least one single-cell sample harbors the reference genotype increases the confidence that the site is not a gSNV; hence, only sites where at least one sample had the reference genotype were included in the analysis. FDR was estimated as the number of sites displaying implausible genotype distributions through the total number of sites displaying plausible and implausible genotype distributions. On raw Monovar output, we applied the recommended filtering [4], including removal of sites overlapping with raw variant calling output of a bulk sample (obtained by FreeBayes), as well as sites present within 10 bases of another site. Parsing putative sSNVs from raw Monovar output yielded an unrealistically high number of sites and a high FDR (Fig. 3a, Additional file 3 Table S2).

To obtain only high confidence genotypes from Monovar output, we applied filters for the genotype quality (GQ). Applying quality filters is a common approach aimed at removing errors in variant calling output [15]. The GQ score is calculated for each predicted genotype, reflecting the probability that the genotype prediction is correct. To compute FDR, we again analyzed sites where a variant call was observed in multiple cells and at least one cell was predicted to be unmutated. Genotypes in individual samples which did not pass the evaluated GQ score cutoffs were defined as unknown. When applying GQ filters, > 99% of sites were filtered out, as compared to when no GQ score filters were applied (Fig. 3b, Additional file 3 Table S2). However, the FDR was comparable regardless of filters for GQ and read depth (DP), when requiring for a variant to be called in > 3 samples (Fig. 3, Additional file 3 Table S2). Following variant calling with Conbase, no further quality filtering is required and called genotypes can be used directly in downstream analysis. Also for Conbase output, we computed FDR by analyzing sites where a variant call was observed in multiple cells and at least one cell was predicted to be unmutated. Here we found that Conbase achieved low FDR, when a variant was required to be observed in > 3 cells (Fig. 3). While the FDR is higher for Monovar compared to Conbase, Monovar detects more sites in absolute numbers when no threshold for genotype quality was applied. This may be explained by the fact that Monovar is not restricted to calling variants in proximity to gSNVs and can thereby detect sSNVs in a larger fraction of the genome as compared to Conbase. Of note, sites displaying a plausible distribution of genotype predictions can occur by chance when genotype data is missing for multiple samples in the dataset due to dropout, as is the case for this dataset.

### Performance evaluation on real world data obtained from in vivo expanded human CD8+ T cells

We next generated a dataset using MDA, a WGA method using the proofreading polymerase Phi29, which is associated with lower error rate and increased amplification efficiency [6]. In accordance with MALBAC data, varying degrees of allelic dropout has been reported for MDA data [12, 16]. To evaluate Conbase on data generated from cells harvested directly from healthy human subjects, we examined CD8^+^ T cell clones that had been expanded in vivo after yellow fever virus vaccination (YFV-17D). Clonally related cells were defined by sequencing of genomically rearranged T cell receptor (TCR) genes. Vaccination triggers the activation of individual naive T cells in lymphoid organs, leading to their expansion to large numbers of effector cells in the lymphoid tissues, which subsequently enter the circulation where they are detectable for at least several months after vaccination [17]. Expanded virus-specific CD8^+^ T cells can be identified and sorted from peripheral blood by labeling cells with fluorescently labeled dextramers which are streptavidin-linked HLA Class I complexes bound to a single viral epitope (Additional file 1 Figure S5) [18]. For this study, we selected CD8^+^ T cells which had responded to a previously identified HLA-B7-restricited viral epitope for YFV (HLA-B7:RPIDDRFGL), which we observed to exhibit a reduced diversity of responding cells relative the dominant HLA-A2 epitope [18, 19]. Single CD8^+^ T cells sorted by fluorescence-activated cell sorting (FACS) from longitudinal peripheral blood samples were amplified by MDA and subsequently screened by PCR against either the TCR α and/or β chains to identify clonally related T cells sharing the same TCR. Libraries from two clones, clone A (seven single cells) and clone B (nine single cells), as well as two unrelated cells (UR) were subjected to whole genome sequencing (Additional file 2 Table S1). As compared to MALBAC data, the percentage of bases covered by reads was higher in MDA data and the error rate was lower (Additional file 1 Figure S6).

For this dataset, we evaluated the performance of Conbase with Monovar and LiRA. When running SCcaller on this dataset, no variant calls passed the defined confidence thresholds recommended to use for SCcaller. This finding is explained by the high amplification bias observed in this dataset, which prevents SCcaller to distinguish true sSNVs from artifacts. Following variant calling with Conbase, no further quality filtering is required, although cutoffs for read depth were evaluated (Additional file 3 Table S2). For Monovar output, we attempted a range of cutoffs for DP and GQ, and the required number of cells in which a variant call was observed, in order to obtain putative sSNVs from Monovar output. Decreased DP cutoffs resulted in increased number of variant sites (Additional file 3 Table S2). In agreement with results from fibroblast data, applying no filters for GQ resulted in an unrealistically high number of variant sites. When applying GQ cutoffs on Monovar output, > 99% of sites were filtered out, as compared to applying no cutoffs for GQ (Additional file 3 Table S2). In contrast to Conbase and Monovar, LiRA does not perform genotyping across the cell population, and variants are called individually per sample. To identify putative clonal somatic variants, we merged output from the individual samples and required that a somatic variant (classified as “PASS” in LiRA output) was observed in > 1 sample (Additional file 3 Table S2).

**Fig. 4:**
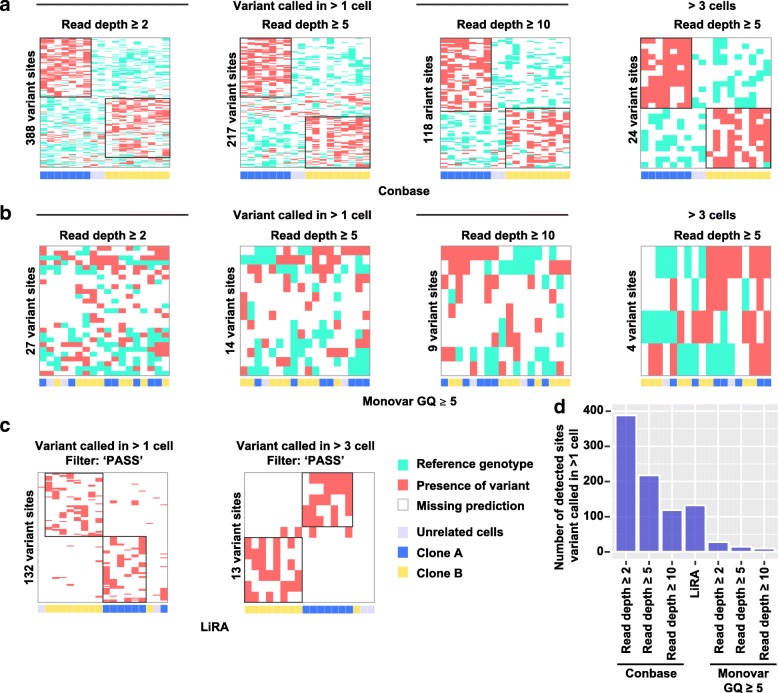
**a**–**c** Hierarchical clustering using genotypes called by Conbase, Monovar, and LiRA to define distances between cells. The obtained clusters are compared to the known clonal relations, shown below each plot. **d** The total number of clonal variant calls, defined as a call being present in at least two cells, obtained by Conbase, LiRA, and Monovar (GQ ≥ 5)

To investigate if variants called by Conbase, Monovar, and LiRA enable separation of clonal populations of cells, we performed unsupervised hierarchical clustering using shared genotype calls in sSNV sites to define distances between cells (Fig. 4). Hierarchical clustering based on genotypes called by Conbase demonstrated unambiguous identification of each T cell clonal population, regardless of read depth cutoffs (Fig. 4a, Additional file 1 Figure S7). For Monovar output, we attempted a range of combinations of filters to parse putative sSNVs from the vcf output. However, no combination of cutoffs for DP or GQ, or when requiring for a variant to be present in increasing number of samples, enabled separation of the clonal populations (Fig. 4b, Additional file 1 Figure S8). The somatic variants predicted by LiRA enabled separation of the clonal populations by hierarchical clustering (Fig. 4a, Additional file 1 Figure S9). However, the total number of sites passing filters was smaller in LiRA output compared to the number of sites called by Conbase with a read depth cutoff ≥ 2 and ≥ 5 (Additional file 3 Table S2).

**Fig. 5:**
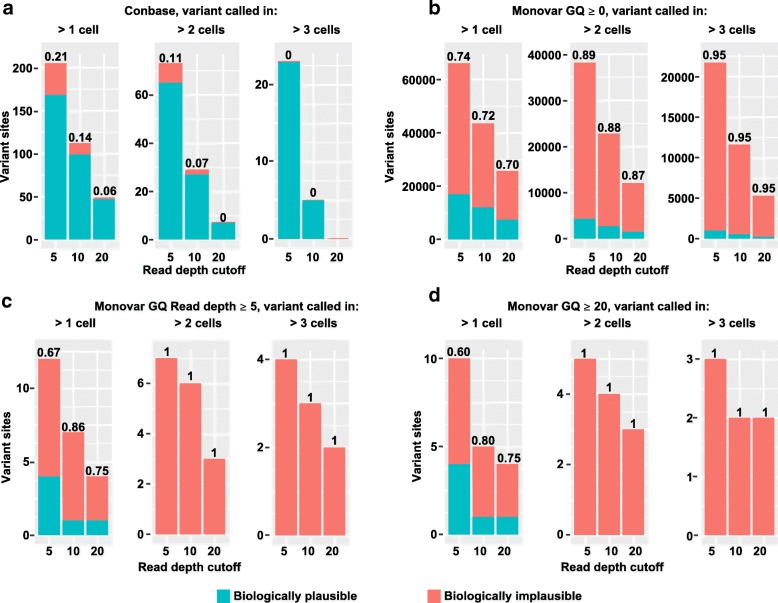
**a**–**d** Biologically plausible and implausible distributions of predicted genotypes in putative sSNV sites called by Conbase and Monovar in clonal populations of T cells. Values above bars represent false discovery rates. Biologically plausible genotype distributions were defined as sites where the variant call is exclusively observed within cells belonging to the same clone. Biologically implausible genotype distributions were defined as sites where the variant call is observed within both clones and at least one cell displayed the reference genotype

To estimate FDR in the T cell dataset, we computed the fraction of sites displaying a biologically plausible and implausible distribution of genotypes in our clonal populations. Estimating FDR for clonal somatic variant calling in this way is only possible in output from Conbase and Monovar, since LiRA does not predict the absence of mutations in unmutated samples. The fraction of sites displaying biologically implausible genotype distributions in the T cell clones was small in Conbase output (Fig. 5a). By default, Conbase filter sites in which at least one sample display conflicting genotypes, with support for both a mutated and an unmutated genotype. When allowing samples with conflicting genotypes in the final output from Conbase, we observed that 90% of sites in which at least one sample displayed conflicting genotypes also displayed a biologically implausible distribution of genotypes (data not shown). In accordance with observations in the fibroblast dataset, we found that, in absolute numbers, Monovar detects more sites than Conbase when no thresholds for GQ is applied (Fig. 5a, b) and that Conbase achieves a low FDR while the FDR is very high for Monovar, regardless of a range of evaluated sets of filters on variant calling output (Fig. 5a–d). These findings support the notion that there is a tradeoff between FDR and the absolute number of detected sSNVs.

### Validation of sSNVs identified by Conbase

**Fig. 6:**
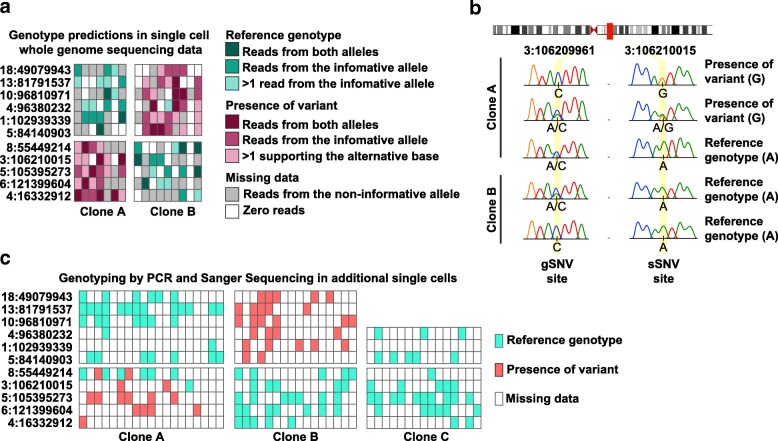
**a** Genotype predictions by Conbase in 11 sSNV sites. Primers spanning sSNVs and gSNVs were designed against these sites for PCR screening and Sanger sequencing. **b**, **c** Sanger sequencing results for the selected sSNV sites in amplified DNA from single CD8+ T cells identified by TCR sequencing as belonging to clones A, B, or an unrelated clone C

We validated a selection of the sSNVs called in T cells by Conbase, through PCR screening of additional MDA libraries generated from single CD8^+^ T cells isolated in parallel and determined to be clonally related by TCR sequence to the cells subjected to high-coverage whole genome sequencing. As a control, we included single CD8^+^ T cells identified as a third clonal population (Clone C, Additional file 2 Table S1) that was not included in the whole genome sequencing experiment. We designed PCR primers flanking regions containing both gSNVs and sSNVs in order to determine whether allelic dropout or amplification bias led to loss of informative alleles in our PCR products. Gel-purified PCR amplicons from each single cell were subsequently subjected to Sanger sequencing to establish the presence or absence of the informative allele, as determined by the gSNV, and the presence of absence of the sSNV called in whole genome sequencing data. Here, we could confirm the presence of sSNVs in cells belonging to the same clones, but never in unrelated cells, providing definitive evidence that the sSNVs called by Conbase represent true somatic mutations in each clonal lineage in vivo (Fig. 6). In the Sanger results, we observed two sSNV sites (3:106210015 and 8:55449214), displaying heterogeneity within the clones, with some cells harboring the reference genotype and some cells harboring the alternative genotype. This could indicate that these two mutations appeared later during clonal expansion.

## Discussion

Conbase is to our knowledge the first available software capable of leveraging phased read data from multiple samples in a dataset, thereby improving confidence in variant calls and genotype predictions in single-cell data. Variant calling and genotyping are based on analysis of observed haplotype concordance, enabling exclusion of false positive variants resulting from alignment artifacts and WGA errors. False negative genotype calls are eliminated through analysis of locus-specific allelic dropout, determined individually per sample. As such, Conbase analysis is not dependent on global estimates of allelic dropout, and genotypes can be predicted in samples exhibiting high rates of allelic dropout. The strength of Conbase is that it considers compatibility among related cells in a population and at the same time uses read phasing. This makes Conbase less sensitive to errors, including PCR, base calling, and alignment artifacts, and genotypes can be predicted at low read depth. One limitation of Conbase is that read phasing restricts the analysis to bases present in the same read or read pair as heterozygous gSNVs, corresponding to ~ 50% of the genome with an average insert size of the sequencing library corresponding to 650 bp.

We have compared Conbase to Monovar, SCcaller, and LiRA, which are variant callers designed to account for errors and biases in single-cell DNA sequencing data [4, 5, 11]. On simulated phased data, comprising sSNV sites displaying both heterozygous (mutated) and homozygous (unmutated) states over the population of cells, Conbase achieved the best performance overall of the tested methods. On simulated data displaying amplification bias, Conbase, Monovar, and LiRA performed comparably in terms of sensitivity, whereas SCcaller performed worse (Fig. 2a). The sensitivity of SCcaller was substantially improved in simulated data exhibiting even read coverage (Additional file 1 Figure S3b).

In simulations, Conbase outperformed Monovar, SCcaller, and LiRA in terms of specificity and FDR, displaying superior ability to detect and exclude artifacts (Fig. 2b, c, Additional file 1 Figure S3c, d). These results are supported by our analysis of real data of clonal populations of cells, where FDR could be estimated using the biological plausibility of the predicted genotype distribution for a detected sSNV among clones. Since only Conbase and Monovar predict both mutated and unmutated genotype states, FDR in clonal somatic variant calling on real data was only computed for these two methods. While Monovar detected more sites in absolute numbers, this was achieved at the expense of a substantially higher FDR (Figs. 3 and 5). The high FDR estimated for Monovar in our simulations and real data is supported by findings reported in another study [5]. The low FDR achieved by Conbase can likely be attributed to its ability to effectively detect and filter false positive variant calls through a combination of read phasing and joint variant calling.

In real data, variants predicted by Conbase and LiRA enabled separation of clonally related populations of single cells from human donors, indicating that the called sSNVs represent true somatic mutations (Fig. 4a, b). Of these two methods, Conbase provided better robustness than LiRA by detecting a higher number of sSNV sites. In sheer numbers, Monovar detected more sites than Conbase and LiRA when no thresholds for GQ were applied (Figs. 3 and 5, Additional file 1 Figure S7–S9). However, the genotypes called by Monovar did not enable separation of the clonal populations (Fig. 4, Additional file 1 Figure S8). Applying filters for GQ on Monovar output did not improve separation of the clonal populations and, moreover, resulted in fewer number of sites passing filters, in comparison to Conbase and LiRA (Figs. 4 and 5, Additional file 1 Figure S7–S9). The failure to identify the clonal populations using genotypes predicted by Monovar may be attributed to the fact that the distinction between true sSNVs and false positives is primarily based on modeling amplification errors, but does not directly account for the extent of ambiguous read mapping (EAL) during variant calling and genotyping. Conversely, the ability of Conbase and LiRA to call clonal variants that enable separation of the clonal cell populations may be explained by the use of read phasing, which is employed by both of these methods and enable exclusion of variants supported by reads derived from discordant alleles. It was surprising that LiRA, while achieving identification of sSNVs that enable separation of clonal populations in real data, exhibited a high FDR in simulated data (Fig. 2c, Additional file 1 Figure S3d). In theory, analysis of the concordance of phased gSNVs provided by SHAPEIT2 [20] and implemented in LiRA should enable exclusion of artifacts present in regions where alignment artifacts occur. One possible explanation is that our simulations could not fully represent the complexity of the effects of alignment artifacts, given that the distribution and frequency of structural variation and low-complexity DNA in the human genome is not well characterized [21]. Possibly, our simulations failed to provide phasing information over larger genomic regions, required for LiRA to detect alignment artifacts in simulated data.

Further investigating the variant calling with Conbase, we were able to experimentally validate that identified sSNVs represented true somatic mutations by PCR screening of additional single cells sorted from the same donor and identified as being clonally related to the two clonal populations used for whole genome sequencing (Fig. 6). We did not detect these variants in any cells isolated in parallel from a third unrelated clone. Indeed, we believe that this approach will provide a useful platform for expanding the analysis to hundreds or thousands of cells using targeted screening after identification of high confidence mutations in single-cell whole genome sequencing data by Conbase.

Lastly, it is interesting to compare how well the different methods predict actual genotypes in different cells in simulated sSNV sites (Fig. 2d, Additional file 1 Figure S3e). Conbase, Monovar, and LiRA performed comparably in terms of correctly predicting heterozygous mutated genotypes, whereas SCcaller predicted fewer true heterozygous mutated genotypes in data exhibiting amplification bias. However, while Monovar detected a higher number of true homozygous unmutated genotypes than Conbase, this was achieved at the expense of a higher number of incorrectly predicted homozygous unmutated genotypes. Notably, Conbase predicted no false unmutated homozygotes. SCcaller and LiRA only predicted the presence of variants, and therefore, no correct or incorrect predictions in homozygous samples were made by these methods. The choice of only predicting the presence of variants conflates homozygous (unmutated) states with failed predictions (missing data) and is a serious drawback for a number of applications, notably phylogenetic analysis.

It is clear that sSNV calling is a balance between the number of variants called and the proportion of false discoveries. For example, Monovar, which do not integrate read phasing, predicts substantially more sSNV sites in absolute numbers than do Conbase (Fig. 1b). However, this is done at the expense of a very high FDR and a very low specificity, meaning that there is a large probability that a predicted variant is erroneously called. Conbase uses phasing information and joint variant calling across a population of related cells to address the FDR problem and calls variants with very low probability of being wrong, but the total number of predicted variants are fewer than that of Monovar. It should be emphasized that Conbase is designed to be used on data from related cells and that it requires a variant to be present in at least two of these cells. LiRA also uses phasing information and provides a certain amount of clonal variants, but suffer from the inability to predict unmutated genotypes. A user’s choice of which variant caller to use will depend on the application of the resulting data. When high-quality variant calls are more important than the sheer number of calls, for example, as in phylogenetic analysis and high resolution lineage tracing at the single-cell level, Conbase is likely to be the best choice. If the aim requires a large total number of variant calls regardless of the abundance of false calls or if the data at hand comprise a single cell or a set of unrelated cells, then Conbase may not be the optimal choice.

## Conclusion

We present Conbase, a new software for somatic variant calling in single-cell data. We show that by exploiting phased read data in multiple cells, Conbase retrieves confident variant calling with low false discovery rate that provide more robustness in downstream analyses.

## Methods

### Algorithm description

Conbase requires whole genome sequencing data from related WGA-amplified single cells and an unamplified bulk sample to predict sSNV sites and genotypes. The identification of clonal somatic variants is unsupervised, such that no prior information about clonal relationships is provided. Conbase takes three inputs: single cell and bulk bam files, a human reference genome in fasta format, and gSNV coordinates to be used for phasing. The gSNV coordinates and gSNV base observations are obtained from vcf output previously generated from variant calling in a bulk sample by FreeBayes (or another variant caller). The analysis is split up in two subprograms: stats and analyze. Stats outputs a json-file with unfiltered variant calls, which is used as input to analyze. The output of analyze are phased filtered variant calls. Conbase variant calling is based on assumptions associated with expected observations in true sSNV sites, including concordance of base observations in independent positions in reads and read pairs within samples and across the dataset (Additional file 1 Figure S1). The assumptions are coded as adjustable parameters with values reflecting how much read observations may deviate from the expected attributes of a true variant site. The parameter values generating the data presented in the current report are specified in parentheses in the algorithm description below.

### Stats

The initial step consists of identifying sites supporting an alternative (potentially mutated) base as compared to the bulk sample. This is done by only considering bases present in the same read or read pair as at least one gSNV using a BAM reader (pysam). The genomic windows in which the analysis will take place are determined by defining the longest distance upstream and downstream of each gSNV, covered by read pairs within the dataset. Because of sequencing errors, amplification errors, or alignment artifacts, more than one alternative base may be observed in the same position, despite a diploid genome. In order to determine the most probable alternative base in a site, we utilize the accumulative information given by all qualified samples in the following way:

Let *R* be the reference base in the bulk and let *b* ∈ *B* = {*A*, *C*, *G*, *T*} ∖ {*R*}. Let ${N}_b^s$ be the read depth of *b* in sample *s* at a given position and let and ${N}_R^s$ be the read depth of *R*.

Let ${V}_b^s$ define the voting result for a base *b* by a sample *s*

$$ {V}_b^s=\left\{\begin{array}{lll}\kern0.5em 1&\ if\ \frac{N_b^s}{N_R^s}\ge \alpha, {N}_b^s+{N}_R^s\ge \beta & \kern0.5em (1)\\ {}& & \\ {}\kern0.5em 1&\ if\ \frac{N_b^s}{N_R^s}<\alpha, {N}_b^s\ge \beta & \kern0.5em (2)\\ {}& & \\ {}\kern0.5em 0&\ otherwise\end{array}\right. $$

where *α* (0.2) and *β* (10x) are parameters for variant allele fraction and total read depth, determining which samples are qualified to vote. In the first case (1), reads from two alleles are observed, displaying sufficient variant allele fraction and total read depth. In the second case (2), the variant allele fraction is not sufficient; however, the sample displays sufficient read coverage supporting the alternative base, which represents another indication for a potentially true variant since amplification bias may result in dropout of the reference base.

Let *V*_*b*_ be the number of samples that voted for an alternative base *b*. If *V*_*b*_ > 0, we may define the most probable alternative base *A* as

$$ A=\left\{\begin{array}{ll}\kern0.5em {argmax}_b\left({V}_b|\forall b\in B\right)& \kern3em if\ \frac{V_b}{\left|V\right|}\ge \gamma, \kern0.75em \left|V\right|\ge \varepsilon \\ {}& \\ {}\kern0.5em undefined& \kern3em otherwise\end{array}\right. $$

where *γ* (0.9) and *ε* (2) define the fraction of samples required to vote for the same base and the number of samples required to vote at a given position, respectively. ∣*V*∣ is the number of samples that were qualified to vote.

### Analyze

The previous processing identified potential sSNV sites in near distance of gSNVs where multiple cells shared strong support for the same alternative base *A*. If we consider the alternative base *A* in the sSNV site in relation to the base observed in a gSNV site in the same read or read pair, we denote it as the following relation:

$$ {X}_{mut}{Y}_{gsnv} $$

where the sSNV site *X*_*mut*_ either displays the same base observed in the reference genome (and the bulk sample) or the alternative base, and *Y* either displays the same base as the reference genome or the alternative base in the gSNV site (obtained from vcf output from variant calling in the bulk sample). For simplicity, we will further denote these relationships as *RR*, *RA*, *AR*, and *AA* where the first letter refers to the observation in the sSNV site and the second letter refers to the observation in the gSNV site (in both cases, we let *R* and *A* denote the reference and the alternative base, respectively). From here on, we will denote a given pair as a *tuple*. Furthermore, a tuple pair, *tp*, denotes the possible combination of tuples on a chromosome pair.

Considering both the maternal and paternal pair, there are only two plausible tuple pairs that could equal a true heterozygous genotype. Likewise, there is only one single type of tuple pair that constitutes a true homozygous genotype. Hence, only three tuples are relevant, namely

$$ \mathrm{Heterozygous}\ \mathrm{genotype}=\left\{ RR, AA\right\},\left\{ RA, AR\right\} $$

$$ \mathrm{Homozygous}\ \mathrm{genotype}=\left\{ RR, RA\Big\}\ \right. $$

Given that we expect a clonal mutation to be present in a subpopulation of the analyzed cells, we can collectively determine the expected coupling representing a true heterozygous genotype, either {*RR*, *AA*} or {*RA*, *AA*}, by analyzing tuple pairs in all the samples that qualifies for voting.

All samples that display reads covering the tuple (i.e., the reads contain the specific gSNV in mind and the sSNV) with a total read depth >*φ* (see individual figures for the examined read depth cutoffs, Figs. 3, 4, and 5, Additional file 1 Figure S7) are allowed to vote if they fulfill the following criteria: Let *tp*_*max*_, *tp*_*min*_ be the tuple pairs with the highest and lowest depth out of the two possible tuple pairs for heterozygous positions. A sample is allowed to vote for *tp*_*max*_ if (i) the ratio between the read depth of *tp*_*max*_ and *tp*_*min*_ < *ρ* (0.01) and (ii) either the ratio for the tuples in *tp*_*max*_ > *λ* (0.1) or if the tuple covering the variant {*AR or AA*} is the maximum out of all tuples for that sample. Out of the entitled samples that voted for their local *tp*_*s*_, the most probable tuple pair across all samples *tp*^∗^ is finalized if at least *κ* (see individual figures for the examined number of samples required to harbor the variant, Figs. 3, 4, and 5, Additional file 1 Figure S7) samples voted and if *tp*^∗^ held a majority of at least *ω*% (90%) of the votes. If the voting samples do not manage to conclude the final tuple pair for a sSNV in connection to a specific gSNV that will dictate across all samples, the allelic origin of the alternative base is simply unknown.

By knowing the designated *tp*^∗^, we also know which of the two alleles we expect to observe the variant on, either {*AA*} or {*AR*}. Consider the following: while we may jump to the conclusion of having found a variant as soon as we observe, e.g., tuple *AA* in a sample, if the *tp*^∗^ actually consists of tuples {*AR*, *RA*}, we actually know for certain that this cannot be a true variant as such observation would be contradictory. Moreover, given this *tp*^∗^, if a sample only display reads supporting the tuple *RA*, we obviously cannot determine the genotype since RA is expected to be observed in both heterozygous and homozygous genotypes.

Let *N*_*x*_ be the read depth of the tuple *x* and *N*_*tot*_ the total read depth for all tuples. Consider the case when *tp*^∗^ = {*RR*, *AA*} and let

$$ {\displaystyle \begin{array}{llll}& {d}_I^{het}& =& \frac{\min \left({N}_{RR},{N}_{AA}\right)}{N_{RR}+{N}_{AA}}\\ {}& {d}_E^{het}& =& \frac{N_{RR}+{N}_{AA}}{N_{tot}}\\ {}& {d}_I^{hom}& =& \frac{\min \left({N}_{RR},{N}_{RA}\right)}{N_{RR}+{N}_{RA}}\\ {}& {d}_E^{hom}& =& \frac{N_{RR}+{N}_{RA}}{N_{tot}}\end{array}} $$

where ${d}_I^x$ is the (internal) ratio for the tuples and ${d}_E^x$ is the (external) ratio for a given tuple pair in relation to all of the tuples for a site from a homozygous and heterozygous perspective. These ratios will be used to determine the confidence in the allelic origin of the sSNV for a given site. The case when *tp*^∗^ = {*RA*, *AR*} is treated analogously.

If *tp*^∗^ is {*RR*, *AA*}, reads displaying *A* in the gSNV site is the informative allele, and reads originating from the informative allele are required for genotyping. If *tp*^∗^ is {*RA*, *AR*}, reads displaying *R* in the gSNV site is the informative allele.

Genotypes are classified as being supported by reads originating from both alleles or supported by reads originating from only the informative allele. This enables genotyping despite 100% allelic dropout, if reads originating from the informative allele are observed.

A genotype determination ${GT}_{SNV}^s$ for any of these two classes of genotypes (either supported by reads originating from two alleles or only supported by reads originating from the informative allele) for a given position in relation to a specific gSNV for a sample *s* will only be possible if there is a given *tp*^∗^ and total read depth of all tuples >*φ* (see individual plots for the examined read depth cutoffs, Figs. 3, 4, and 5, Additional file 1 Figure S7). If these initial conditions are met, ${GT}_{gSNV}^s$ is computed as follows:

$$ {GT}_{gSNV}^s=\left\{\begin{array}{ll}\kern0.5em {het}_{C1}& \kern0.15em if\ {d}_E^{het}\ge \theta, {d}_I^{het}\ge \tau \\ {}& \\ {}\kern0.5em {\mathit{\hom}}_{C1}& \kern0.15em if\ {d}_E^{hom}\ge \theta, {d}_I^{hom}\ge \tau\ \\ {}& \\ {}\kern0.5em {het}_{C2}& \kern0.15em if\ {d}_I^{het},{d}_I^{hom}>\tau, {d}_E^{het}\ge \theta, {d}_E^{hom}<1-\theta \\ {}& \\ {}\kern0.5em {\mathit{\hom}}_{C2}& \kern0.15em if\ {d}_I^{het},{d}_I^{hom}<\tau, {d}_E^{hom}\ge \theta, {d}_E^{het}<1-\theta \\ {}& \\ {}\kern0.5em conflict& \kern0.15em if\ conditions\  met\  for\ {het}_{C1},{\mathit{\hom}}_{\mathrm{C}1}\\ {}& \\ {}\kern0.5em conflict& \kern0.15em if\ {tp}_s\ne {tp}^{\ast}\end{array}\right. $$

where *τ* (0.1) is the internal ratio parameter and *θ* (0.9) is the external ratio parameter.

The reads covering the sSNV may cover multiple gSNVs, which is why a collectively decided final genotype can be defined by letting all qualified $G{T}_{SNV}^s$ vote for the final genotype. $G{T}_{max}^s$ is therefore the genotype with the most votes. Let *N*_*max*_ be the number of gSNVs that voted for $G{T}_{max}^s$, and *N*_*vot*_ is the number of gSNVs that were qualified to vote (*tp*^∗^ is defined for this gSNV). Let *N*_*tot*_ be the number of gSNVs that were present in the same read or read pairs as the sSNV and let *GT*^*s*^ be defined as

$$ {GT}^s=\left\{\begin{array}{ll}\kern0.5em {GT}_{max}^s&\ if\kern0.75em \frac{N_{max}}{N_{vot}}\ge \psi, \frac{N_{vot}}{N_{tot}}\ge \sigma \\ {}& \\ {}\kern0.5em conflict&\ if\kern0.75em \frac{N_{max}}{N_{vot}}<\psi \end{array}\right. $$

where *ψ* (0.9) is the fraction of gSNVs voting for the same genotype and *σ* (0.9) is the fraction of gSNVs qualified to vote for a genotype.

If *N*_*vot*_ = 0, any available read information is used to infer genotypes for samples displaying insufficient read depth. Support for an unmutated genotype is defined as either when the *tp*^∗^ = {*AA*, *RR*} and the sample displays a read depth supporting the tuple *RA* > *χ* (1x) or when the *tp*^∗^ = {*AR*, *RA*} and the sample displays a read depth supporting the tuple *RR* > *χ*, resulting in *GT*^*s*^ = *hom*_*C*3_. If there is no support for an unmutated genotype and the sample displays a read depth supporting the alternative base with a value >*ξ* (1x), then *GT*^*s*^ = *het*_*C*3_. Samples displaying support for both a mutated and an unmutated genotype are considered *GT*^*s*^ = *conflict*.

In the current report, zero samples displaying conflicting genotypes were allowed per site. A common artifact in variant calling output is regions with clusters of false positive mutations, correlating with areas in the genome regions with poor mappability. In the current report, a maximum of one mutation per kilobase was allowed.

### Sequence alignment and data processing

Following whole genome sequencing and demultiplexing, the reads were trimmed from Illumina adapters using Cutadapt [22]. WGA adapters were trimmed from MALBAC amplified samples using Cutadapt [22]. Read pairs were aligned to the human genome (human g1k v37 with decoy) using Burrows-Wheeler aligner (BWA-MEM) [23]. Processing of the mapped reads and sequencing data quality evaluation was performed using Picard Tools and FastQC. Read processing included removal PCR duplicates, optical duplicates, and reads with a mapping quality below 2, including multimappers. Indel realignment was performed with GATKs IndelRealigner [24]. Variant calling was performed using FreeBayes with default settings [13].

### gSNV filtering

Following variant calling in bulk samples using FreeBayes, variants were filtered by vcffilter (https://github.com/vcflib/vcflib) to identify gSNVs. False gSNVs will result in false variant calls in the downstream analysis with Conbase. As such, we applied stringent filters on the vcf output from FreeBayes. With an average read depth of ≈ 40x in our bulk samples, we estimated a conservative maximum depth threshold of 55x at any position, as

$$ d+2.5\sqrt{d}, $$

where *d* is the average read depth across the genome.

We filtered variants on autosomes with a 15–55-fold read depth, quality score above 10 (QUAL), and reads originated from both strands (SAF and SAR); at least two reads were balanced on each side of the site (RPR & RPL), and the alternative allele observation count was required to range between 20 and 80% (AO). The variants were further filtered to remove gSNVs present in suspected erroneous regions in the reference genome. This was done by running a separate script included in the Conbase package, which screens the bulk bam file around each gSNV, and excludes gSNVs present within a 1-kb window containing > 10 “heterozygous” positions. A heterozygous position was defined as a position where > 10% of the reads supported a non-reference base. In the fibroblast donor (donor 1), 1,634,933 gSNVs passed filters. In the T cell donor (donor 2), 1,789,830 gSNVs passed filters. Approximately 70% of gSNVs are present within 1200 bases of another gSNV (Additional file 1 Figure S2). Given the observed distribution of gSNVs in the two donors, ~ 50% (unique) genomic bases are present within 650 bases of a gSNV and can thus be phased with an average sequencing library insert size of 650 bp (Fig. 1b, Additional file 1 Figure S2).

### Generation of simulated data

We here provide a summary description of the simulations used to generate reads data for a set of loci in a population of cells. For a more formal and detailed description, please refer to Additional file 4 We aimed to make our simulation as similar to real experimental conditions as possible, and we therefore based our generative model on experimental bulk DNA sequencing data from two human cell populations, the CD8^+^ T cells, and the primary human fibroblast cell line C5RO (normal) used in our analyses of real experimental data. We identify a set of loci comprising two sites *G* and *S* such that for each locus, (1) *G* is a heterozygous gSNV in both the CD8+ T cell data and the fibroblast data. (2) *S* is a heterozygous gSNV in the CD8+ T cell data, but is homozygous for the reference allele in the fibroblast data. (3) *G* and *S* are on the same chromosome and are situated 11–50 bp apart. In the simulations, we let *G* represent a gSNV, while *S* represents a potential sSNV. We then collect all allele-specific reads from the CD8+ T cell data covering *S* and *G* for the different loci, into one read set for each allele and similarly for the fibroblast data. We refer to the pairs of allele-specific read sets from the CD8+ T cell data and from the fibroblast data as *hetReads* and *homReads*, respectively. Moreover, to be able to simulate realistic numbers of reads from different alleles, we obtain allele-specific read coverage distributions, *d*_*COV*_ from single-cell DNA sequencing data from the CD8+ T cell population. Here, we sampled coverage distributions in sites where at least 12 cells were covered by reads.

We now describe the generative model and the input variables used in the simulations. Read data is generated from a set *L* of loci in a population *C* of cells. A clonal population structure of *C* is modeled as a simplified tree *T* comprising two clones of 10 cells each. For a locus *l*, the parameter *snv*_*l*_ determines if *l* is a sSNVs, For a locus, *l*, if *snv*_*l*_ = 1, we set *S* as a sSNV, displaying heterozygote variants (with reads sampled from *hetReads*) in all cells of a randomly sampled clone from *T* (remaining cells are homozygous for the reference state), while if *snv*_*l*_ = 0, all cells are homozygous (with reads sampled from *homReads*) for the reference state. Except in the case of alignment errors (EAL) (see below), we set *snv*_*l*_ = 1 for all loci.

The input parameter *p*_*EAL*_ determines the probability of EAL, by generating a variable *eal*_*l*_, such that *eal*_*l*_ = 1 with probability *p*_*EAL*_; otherwise, *eal*_*l*_ = 0. For any locus *l* with *eal*_*l*_ = 1, we include an additional “paralogous” locus *l*^’^ with the opposite state, from which reads may also be sampled. Notice that in the case of EAL, there is generally no way to know whether the reads from *l* or *l*^’^ are the correct ones. To simplify evaluation, we arbitrarily choose to always simulate an EAL locus *l* such that *snv*_*l*_ = 0. Hence, reads for *l* are sampled from *homReads* and those for *l*^’^ from *hetReads*. Notice that the opposite case would yield the same read distribution.

The input parameter *p*_*DO*_ determines the frequency of allelic dropout among cells at a locus. For each cell *c* and locus *l*, it generates a variable *do*_*c*, *l*, *a*_ where *a* is an allele of either *l* or *l*^’^ (the latter only if *eal*_*l*_ = 1), such that *do*_*c*, *l*, *a*_ = 1 with probability *p*_*DO*_; otherwise, *do*_*c*, *l*, *a*_ = 0. Only alleles *a* for any allele *l* and *l*^’^ in *c*, such that *do*_*c*, *l*, *a*_ = 0, are valid read sampling (i.e., *do*_*c*, *l*, *a*_ = 1 prevents all read sampling from that allele).

Finally, we generate reads; for each cell *c* ∈ *C* and for each valid allele of each *l* and *l*^’^, as determined by *eal*_*l*_ and *ado*_*a*_, we randomly sample *N* reads covering *G* and *S* from the corresponding read set determined by *snv*_*l*_, where *N* is sampled randomly and *i.i.d.* from *d*_*COV*_. All reads from individual cells are stored in separate bam files, which are then used as input to the tested methods.

We performed two different experiments with different statistics being recorded. In the first experiment, we focused on prediction of sSNVs in the population of cells, where we consider a sSNV as predicted if it is found in two or more cells. Here, we simulated read data, as described above, using all value combinations of *p*_*EAL*_ ∈ {0.1, 0.2, …0.9} and *p*_*DO*_ ∈ {0.1, 0.2,  … , 0.9}. We recorded loci correctly predicted as sSNVs (“true positives;” TP), correctly predicted not being sSNVs (“true negatives;” TN), incorrectly predicted as sSNVs (“false positives;” FP), and incorrectly predicted as not being sSNVs (“false negatives;” FN). Using these definitions, we calculated sensitivity (TP/(TP + FN)), specificity (TN/(TN + FP)), and FDR (FP/(FP + TP)). In the second experiment, we focused on genotype prediction in individual cells in sSNV sites. Hence, we set *p*_*EAL*_ = 0, but used the same range of *p*_*DO*_ values as in experiment 1. Here, we recorded loci correctly predicted as heterozygous (true heterozygous), correctly predicted as homozygous (true homozygous), incorrectly predicted as heterozygous (false heterozygous), incorrectly predicted as homozygous (false homozygous), and finally loci where a genotype was not predicted (no prediction—for generated heterozygous or homozygous genotypes, respectively). Since one of the tested methods, SCcaller, is sensitive to uneven read coverage (personal communication with authors of SCcaller), we performed additional simulations, where we repeated experiments 1 and 2, but instead of using the empirical *d*_*COV*_, we enforced a coverage of 30 reads for each allele in all simulated loci.

### Clonal human fibroblast isolation and analysis

Single cells isolated from a primary human fibroblast cell line C5RO (normal) were expanded in vitro on a Leica frame slide. Clonally related cells (determined by time-lapse movie recording) were isolated by LCM. Eleven cells from clone1, three cells from clone2, and two unrelated cells were next subjected to WGA using MALBAC (Yikon Genomics). Samples were individually inspected using a bioanalyzer (Agilent), and library preparation was done using KAPA HTP Library Preparation kit Illumina Platform (KR0426,KAPABIOSYSTEMS) and whole genome sequencing. The cells belonging to clone 1 were sequenced to an average depth of 15x. The single cells belonging to clone 2 and unrelated cells were sequenced to an average depth of 10x. An unamplified bulk sample from the same primary cell line was sequenced to an average depth of 40x.

### T cell sample preparation and cell sorting

Study participants were recruited into an ongoing study to monitor immune responses to the yellow fever virus vaccine YFV-17D (approved by the Regional Ethical Review Board in Stockholm, Sweden: 2008/1881-31/4, 2013/216-32, and 2104/1890-32). A female subject was identified based on being positive for HLA-B7 and having a detectable T cell response to a minor peptide (RPIDDRFGL) presented by HLA-B7. Cryopreserved peripheral blood mononuclear cell (PBMC) samples taken at days 10, 30, and 148 post-vaccination were thawed at 37 **°**C and quickly washed in FACS buffer (PBS with 2% BSA/2 mM EDTA). Negative selection with magnetic beads was performed for each sample to purify CD8^+^ T cells (Miltenyi Human CD8 Negative Selection kit, 130-096-495). Purified CD8^+^ T cells were first incubated with an HLA-B7/RPIDDRFGL dextramer conjugated to Alexa fluor 647 (Immudex) for 15 min. Cells were subsequently incubated with a panel of antibodies to identify live CD3^+^CD8^+^ T cells (CD3–Alexa Fluor 700 (UCHT1, BD Biosciences), CD8-APC-Cy7 (SK1, BD Biosciences), CD4-PE-Cy5 (RPA-T4, eBioscience), CD14–Horizon V500 (MΦP9, BD Biosciences), CD19–Horizon V500 (HIB19, BD Biosciences), and Live/Dead Fixable Aqua Dead Cell staining kit (Invitrogen, L34957)). Live, lineage-negative CD3^+^CD8^+^Dextramer^+^ cells were sorted into 96 well PCR plates (Thermo Scientific, AB-0800) containing lysis buffer (200 mM KOH, 40 mM DTT, 5 mM EDTA). Single cells were incubated on ice for 10 min in lysis buffer; after which, neutralization buffer (400 mM HCL, 600 mM Tris-HCL pH 7.5) was added followed by an additional 10-min incubation on ice. Lysed cells were subsequently stored at − 80 **°**C until amplification reactions were performed.

### WGA by MDA

Lysed single T cells were subjected to multiple displacement amplification (MDA) as previously described [25]. A mixture containing dNTPs (Invitrogen, 2 mM), random hexamer primers with 3′ thiophosphate linkers (5′-dNdNdNdN*dN*dN-3′, IDT (50uM)), and repliPHI polymerase (40 U) in phi29 reaction buffer (Epicenter) was added to each well to bring total volume to 20uL. Cells were incubated at 30 **°**C for 10 h followed by a 3-min incubation at 65 **°**C to inactivate the phi29 polymerase. The resulting libraries were diluted in H_2_O to 50uL and concentrations of double-stranded DNA were measured (Qubit, Broad Range dsDNA kit).

### Identification T cell receptors from single-cell MDA material

We adopted a previously published method [26] so that we could screen large numbers of single-cell libraries to identify clonally related T cells by TCR rearrangements. Approximately 100 ng of amplified DNA was taken from each sample and touchdown PCR (Tm: 72 to > 55 **°**C) was performed using a panel of primers designed upstream of each variable region and downstream of the joining regions for the human TCR α or β chain locus (Additional file 5 Table S3). A dilution of each reaction was subsequently used to perform a second, nested-touchdown PCR with internal primers designed against each variable and joining region of the human TCR α or β chain locus. The internal primers contained handles which were used to index each well for the 96-well plate so that they could be pooled into a single reaction. Each plate was then prepared according to the Truseq (Illumina) protocol for sequencing on an Illumina Miseq (2 × 150bp reads). After demultiplexing of Illumina sample indexes, the reverse read (R2, 150bases) Fastq file was converted to Fasta format. Identical sequences were clustered using the FASTX-Toolkit (http://hannonlab.cshl.edu/fastx_toolkit/) FASTA Collapser. Then, sequences were sorted by our 96-well indexes using the FASTX barcode splitter, and the first 44 bases were finally trimmed off using the FASTA trimmer to facilitate downstream sequence analysis. Because the internal primers targeting the joining regions were within 50 bp of the CDR3 region of the TCR, it was possible to identify clonal T cells based on shared CDR3 nucleotide sequences. All samples were individually analyzed using the IMGT database to identify the CDR3 sequence [27].

### Selecting high-coverage libraries for Illumina sequencing

Clonal T cells were grouped, and high-quality libraries were identified using a panel of chromosome-specific PCR primers as described previously [10]. High-quality T cell libraries were considered to be samples with detection at the majority of loci and were subsequently processed using a PCR-free TruSeq library preparation kit (Illumina) and sequenced with a HiSeq X using a theoretical coverage of 30x per sample (SciLifeLab, Karolinska Institute).

### Screening related clonal T cells by Sanger sequencing

Single-cell libraries that were included in the original screening which matched clones A or clone B were identified to be used for verifying selected mutants (summarized in Additional file 6 Table S4). An additional clone (Clone C (TCRα: CAAHSPYSGNTPLVF, TCRβ: CASSSGTAYNEQFF) was used as a control to determine whether mutations could be found as artifacts in unrelated T cells. Primers were designed to span both the gSNV, and the putative variants and samples were subjected to 35 cycles of PCR (Tm: 67 **°**C) (PCRBIO HiFi Polymerase, PCR Biosystems) yielding approximately 1000 bp amplicons (Additional file 6 Table S4). Additionally, primers contained handles (similar to those used for TCR screening) so that secondary amplification cycles could be used to index samples if necessary. Amplified samples were analyzed by gel electrophoresis, and bands were excised for DNA isolation (Nucleospin Gel Clean Up, Techtum). Gel-purified DNA samples were sent for Sanger sequencing (KI Gene Facility, CMM, Karolinska Institute) using primers specific for the universal handle incorporated onto each Forward primer (Additional file 6 Table S4). Sanger sequencing results were analyzed visually using the software package 4peaks and are summarized in Additional file 6 Table S4.

### Comparisons of single-cell variant calling algorithms

Monovar was run on single T cell amplified with MDA with default settings including consensus filtering. From the raw Monovar output, sites present within 10 bp of another site were removed. Sites overlapping with raw variants called in an unamplified bulk sample by FreeBayes were removed. Potential sSNVs were filtered on autosomes by requiring that at least two samples shared a variant (0/1 or 1/1) while at least one sample displayed the reference genotype (0/0). Samples in the same site which did not pass these cut offs were assigned an unknown genotype. We attempted to call variants in real data using SCcaller [5]. The rate of amplification bias observed in the T cell dataset and the Fibroblast dataset resulted in eta-values that were too low to enable distinction between true mutations and artifacts (personal communication with authors of SCcaller [5]). Thus, we did not move forward with variant calling using the SCcaller. LiRA [9] was run with default settings, with minor fixes to allow us to run it on our server. dbSNP b151_GRCh37p13 was used and 1000 genomes haplotype reference panel from https://mathgen.stats.ox.ac.uk/impute/1000GP_Phase3.tgz. Prior to LiRA the bam files were processed with GATK Haplotype caller with settings (--emitRefConfidence GVCF --variant_index_type LINEAR --variant_index_parameter 128,000). Joint variant calling was done for bulk and single cells using GATK GenotypeGVCFs.

For simulation data, a Snakemake pipeline was used to run all the four methods (Conbase, Monovar, SCcaller, and LiRA), see https://github.com/joannahard/Genome_Biology_2019. All methods were run as described above for real data. For LiRA however, some fixes to the code were required, as the program could not handle chromosomes with no sSNVs. Hence, checkpoints were added at several points in the code and chromosomes with missing data were omitted at subsequent steps. In addition, our simulations did not involve larger regions with surrounding gSNVs, so we created a bulk with all reads 10Kb upstream/downstream of the gSNV and sSNV of interest and borrowed phasing information of the gSNV of interest to surrounding gSNVs from that bulk. The time to run these methods on our simulated data (20 cells for each of 100 different parameter combinations) using 50 cpus in parallel was as follows: 44 min for Monovar, 128 min for SCcaller, 129 min for Conbase, and 63 h for LiRA, not including preprocessing with GATK or Freebayes for LiRA and Conbase, respectively. The SHAPEIT2 [20] step in LiRA is very time-consuming, hence the long run times.

### Hierarchical clustering

Clonal somatic variants called by Conbase, Monovar, and LiRA in real data were used to define distances between cells. For Conbase and Monovar, distances between cells were defined as unknown if no shared sites were detected. For shared sites, the distance was decreased with − 1 for each site where cells have the same call (mutated or non-mutated) and increased with + 1 for sites where the cells have different calls. Since LiRA only predicts the presence of variants, the distance was decreased with − 1 if cells shared a variant; otherwise, the distance was defined as not available. The distance matrix was then clustered using standard hclust with the distance “ward.D2.” For Monovar matrices with more than 45 K sites (no applied GQ filtering), 45 K randomly selected sites were included in the clustering analysis.

### Conbase output

The final output from Conbase includes a tsv file with phased variant calls and a complementary interactive html file where genotype predictions are color coded based on presence or absence of mutation, presence or absence of allelic dropout, and read depth support as well as summarized statistics about concordance of base observations in phased reads in the predicted variant sites for each sample.

### Software

Conbase is implemented in python and is only dependent on the pysam module. Conbase is licensed under the MIT license.

## Additional files

Additional file 1: Supplementary figures. **Figure S1-S9.**, including figure legends. (PDF 3413 kb)

Additional file 2: **Table S1.** (XLSX 11 kb)

Additional file 3: **Table S2.** (XLSX 13 kb)

Additional file 4: Simulation model. A detailed description of the model used for generating the simulated data included in this study. (PDF 117 kb)

Additional file 5: **Table S3.** (XLSX 47 kb)

Additional file 6: **Table S4.** (XLSX 48 kb)

Additional file 7: Review history. (DOCX 87 kb)

## Abbreviations

DP: Read depth field in Monovar variant calling format output
EAL: Alignment error, caused by ambiguous read mapping
FACS: Fluorescence-activated cell sorting
FDR: False discovery rate
GQ: Genotype quality field in Monovar variant calling format output
gSNV: Germline single nucleotide variant
*i.i.d.*: Independent and identically distributed
MALBAC: Multiple annealing and looping-based amplification cycles
MDA: Multiple displacement amplification
sSNV: Somatic single nucleotide variant
WGA: Whole genome amplification
