# Supplementary material for: Conbase: a software for unsupervised discovery of clonal somatic mutations in single cells through read phasing
Source: Genome Biol. 2019 Apr 1;20:68. doi: 10.1186/s13059-019-1673-8 (PMC6444814; doi:10.1186/s13059-019-1673-8)
Supplement: Supplementary file 1 — Supplementary figures. Figure S1-S9., including figure legends. (PDF 3413 kb) [file 13059_2019_1673_MOESM1_ESM.pdf]

Fig. S1

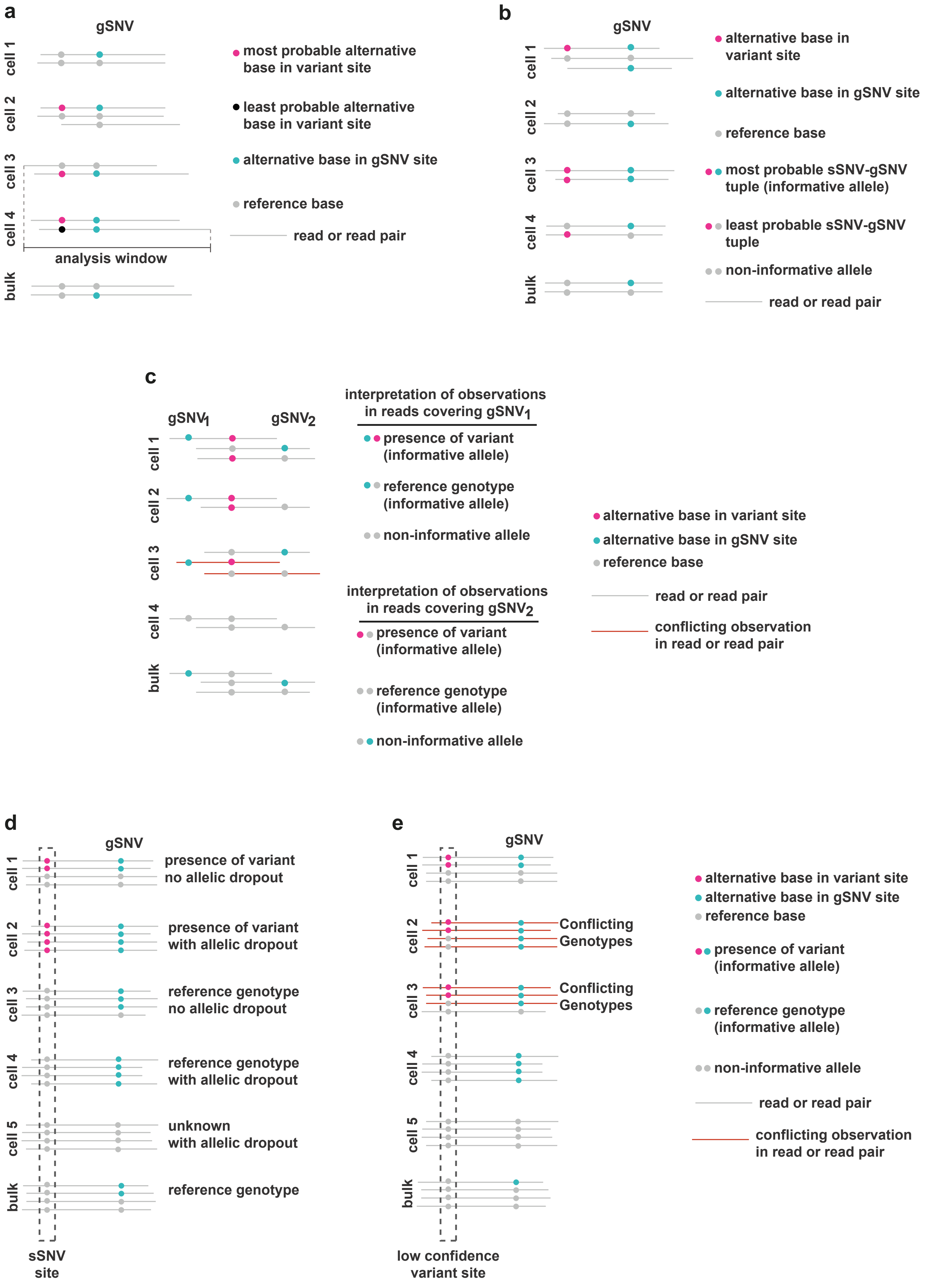
a
gSNV
cell 1
cell 2
cell 3
cell 4
bulk
analysis window
most probable alternative base in variant site
least probable alternative base in variant site
alternative base in gSNV site
reference base
read or read pair
b
gSNV
cell 1
cell 2
cell 3
cell 4
bulk
alternative base in variant site
alternative base in gSNV site
reference base
most probable sSNV-gSNV tuple (informative allele)
least probable sSNV-gSNV tuple
non-informative allele
read or read pair
c
gSNV1
gSNV2
cell 1
cell 2
cell 3
cell 4
bulk
interpretation of observations in reads covering gSNV1
presence of variant (informative allele)
reference genotype (informative allele)
non-informative allele
interpretation of observations in reads covering gSNV2
presence of variant (informative allele)
reference genotype (informative allele)
non-informative allele
alternative base in variant site
alternative base in gSNV site
reference base
read or read pair
conflicting observation in read or read pair
d
gSNV
cell 1
cell 2
cell 3
cell 4
cell 5
bulk
presence of variant no allelic dropout
presence of variant with allelic dropout
reference genotype no allelic dropout
reference genotype with allelic dropout
unknown with allelic dropout
reference genotype
sSNV site
e
gSNV
cell 1
cell 2
cell 3
cell 4
cell 5
bulk
Conflicting Genotypes
Conflicting Genotypes
low confidence variant site
alternative base in variant site
alternative base in gSNV site
reference base
presence of variant (informative allele)
reference genotype (informative allele)
non-informative allele
read or read pair
conflicting observation in read or read pair

Fig. S1. The Conbase algorithm. (a) The genomic windows in which the analysis will take place are determined by defining the longest distance upstream and downstream of each gSNV, covered by read pairs within the dataset. Within the genomic windows, bam files are screened for the presence of alternative bases. Due to sequencing errors, amplification errors and alignment artifacts, multiple alternative bases may be observed in the same site. The most probable alternative base in putative variant sites is determined by a majority vote, taking observations within samples and across samples into account. (b) For all reads and read pairs, the base observations in putative variant sites and gSNV sites are saved as tuples, to be used in the subsequent analysis. The allelic origin of the alternative base in putative variant sites, is determined by analyzing the observed tuples within samples and across the dataset. The allele determined to harbor the variant in mutated samples is the informative allele. The non-informative allele displays the same base observations in the variant site and gSNV site in both mutated and unmutated samples. (c) All gSNVs present in the same read or read pair as putative variants, contribute to genotype predictions. (d) True somatic mutations are present on the same allele in mutated samples (cell 1, cell 2), representing the informative allele. Presence or absence of mutations is determined despite allelic dropout in samples displaying reads originating from the informative allele (cell 2, cell 4). The genotype is unknown in samples only displaying reads from the non-informative allele (cell 5). (e) Sites in which any sample display conflicting genotypes (cell 2, cell 3) are by default filtered out as low confidence variant sites. Conflicting genotypes display support for both mutated and unmutated genotypes, and result from alignment artifacts or amplification errors.

Fig. S2

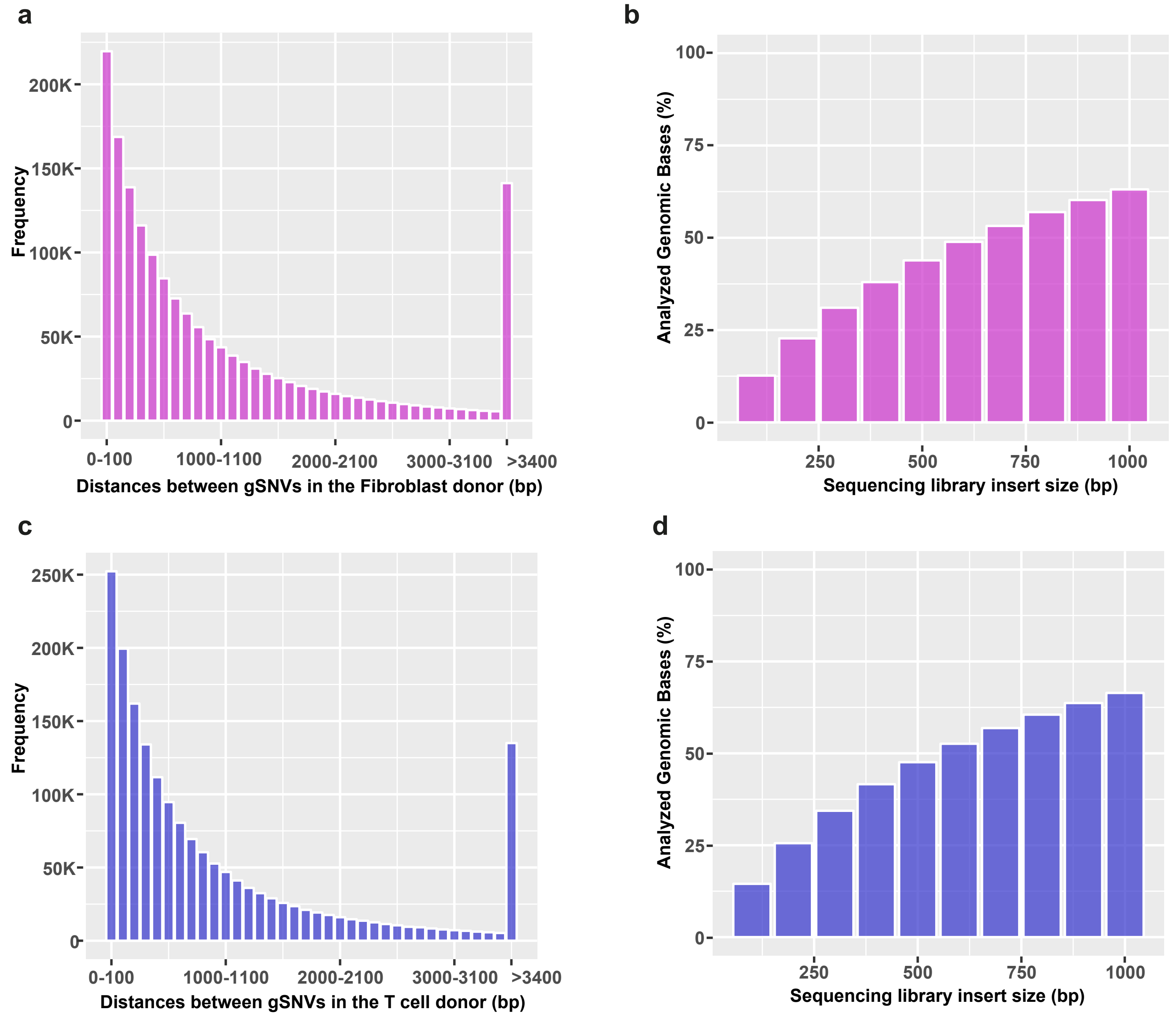

Fig. S2. (a) The distribution of distances between gSNVs in the fibroblast donor. (b) The fraction of unique genomic positions present within 100-1000 bases of gSNVs in the fibroblast donor, representing positions that are analyzable by Conbase, with regards to the insert size of the sequencing library. Only genomic positions covered by at least 1 read in an unamplified bulk sample sequenced at 40x coverage were considered. (c) Distribution of distances between gSNVs in the T cell donor. (d) The fraction of unique genomic positions present within 100-1000 bases of gSNVs in the T cell donor, representing positions that are analyzable by Conbase, with regards to the insert size of the sequencing library. Only genomic positions covered by at least 1 read in an unamplified bulk sample sequenced at 40x coverage were considered.

Fig. S3

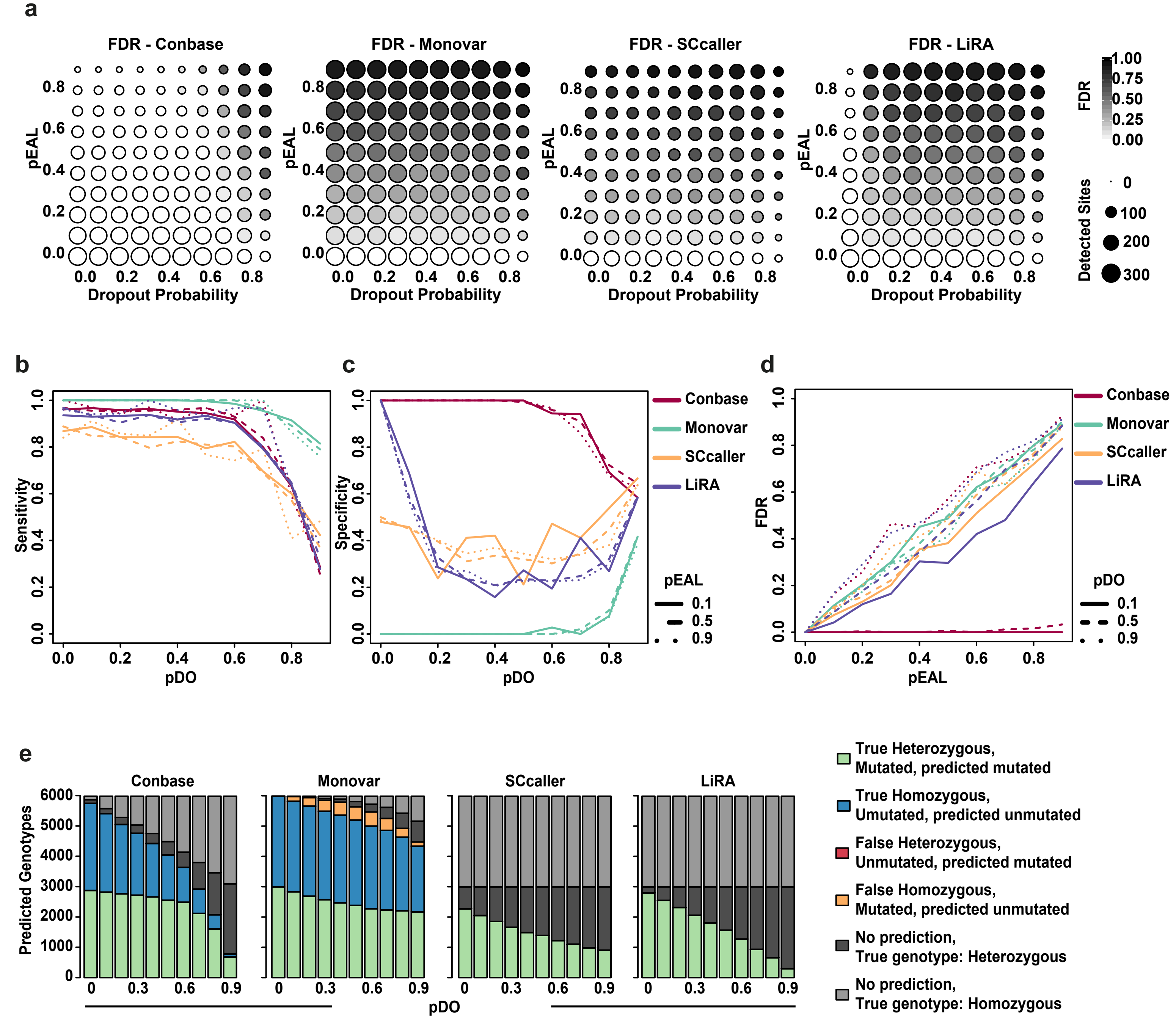

Fig. S3. (a) False discovery rate (FDR) and the total number of detected sites by Conbase, Monovar, SCcaller and LiRA at increasing probability of EAL in simulated data. (b-d) The accuracy of variant calling was evaluated for Conbase, Monovar, SCcaller and LiRA, reflecting the ability of the methods to detect clonal sSNV loci in a population of cells in simulated data with a flat read depth, corresponding to 30x across all sites. The sensitivity (b) and specificity (c) of Conbase, Monovar, SCcaller and LiRA to detect clonal mutations in at least 2 cells in simulated data, at increasing dropout probabilities (pDO) and at different levels of alignment artifact probabilities (pEAL). (d) FDR of Conbase, Monovar, SCcaller and LiRA when detecting clonal mutations in simulated data at increasing alignment artifact probabilities (pEAL) at different levels of dropout probabilities (pDO). (e) The accuracy of genotyping was evaluated for Conbase, Monovar, SCcaller and LiRA, reflecting the ability of the methods to correctly predict genotypes in each sSNV loci and each cell at increasing dropout probabilities (pDO) in simulated data with a flat read depth, corresponding to 30x across all sites. In each simulation, representing one bar, the true genotype in 50% of the samples were heterozygous, representing samples harboring a sSNV. The remaining samples were homozygous for the reference allele, representing the ancestral unmutated state.

Fig. S4

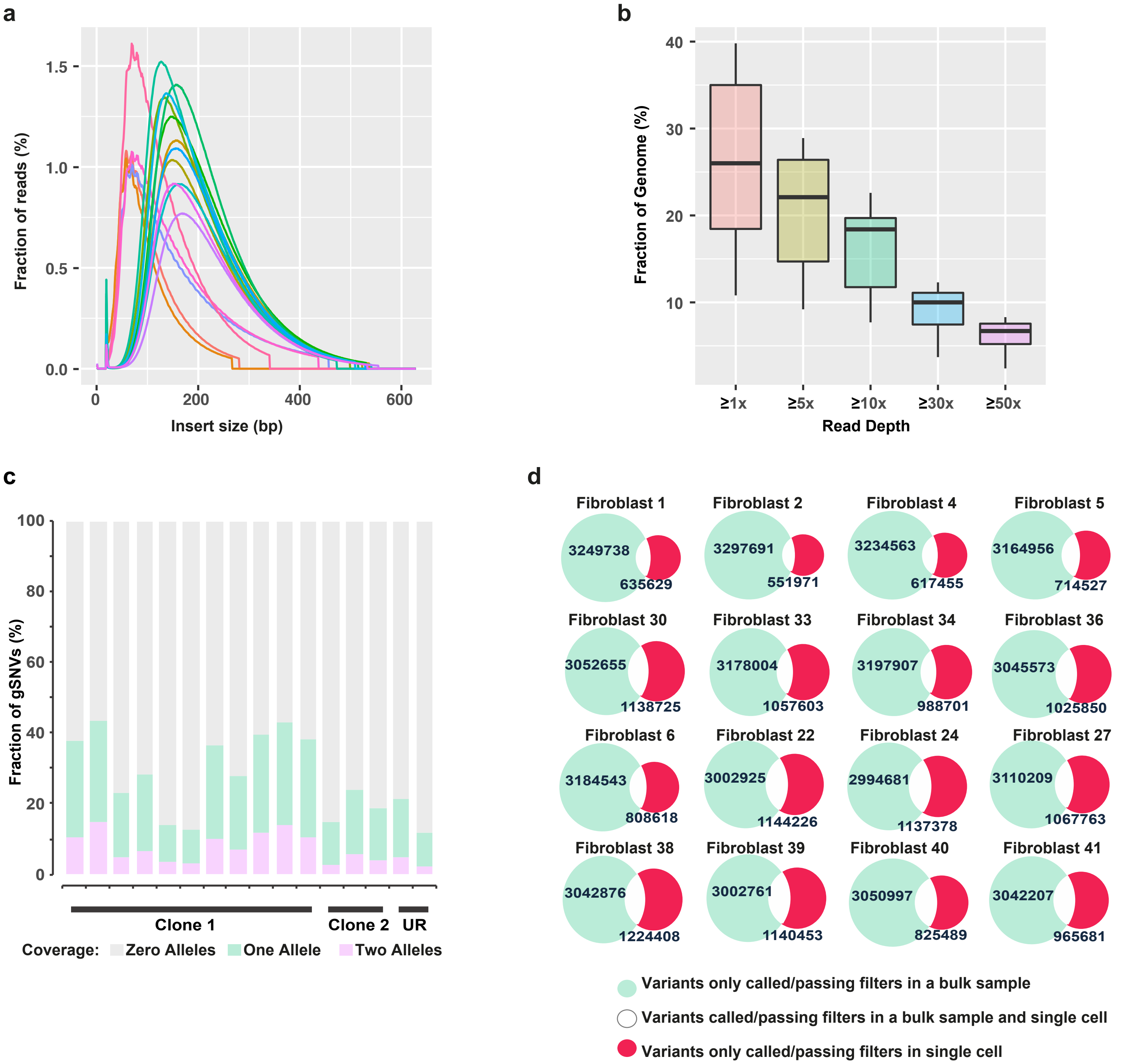

Fig. S4. Data quality statistics in MALBAC-amplified single fibroblasts (a) Distribution of insert sizes in sequencing libraries from single fibroblasts. (b) The fraction of the genome covered by at least 1, 5, 10, 30 and 50 reads per single fibroblast sample (c) Amplification efficiency in single fibroblasts belonging to either of two clones (1 and 2) or to unrelated cells (UR). Amplification efficiency in single fibroblasts was estimated by analysis of read coverage in heterozygous gSNV sites, where reads originating from zero alleles represent locus dropout, reads originated from one represent allelic dropout, and reads originating from two alleles represent sites with full coverage (no allelic dropout). Only reads and bases with a mapping quality and base quality equal or greater than 20 were considered. (d) Overlap of variants called by Freebayes, in an unamplified bulk sample and single fibroblasts. Only variant calls supported by at least 20 reads with a GQ score of at least 20 were considered.

Fig. S5

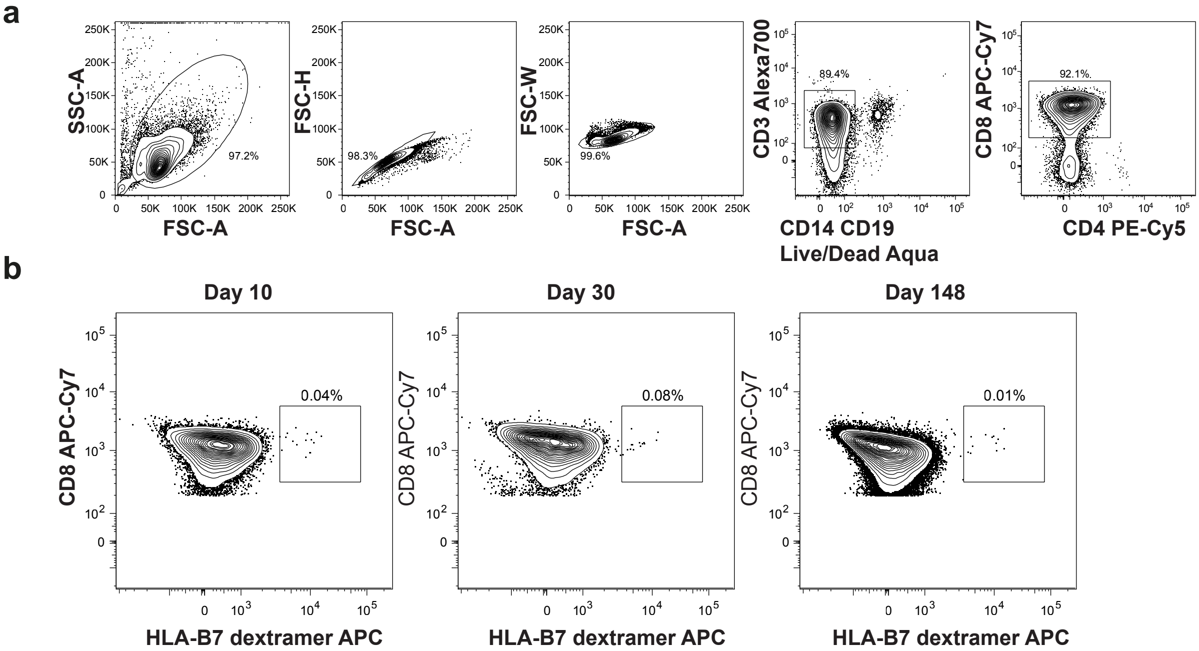

Fig. S5. Gating strategy for sorting single CD8+ T cells from vaccinated healthy donor blood. (a) Basic strategy to isolate single CD8+ T cells includes gating on size distribution (FSC vs SSC) followed by singlet gating using FSC area versus height and FSC area versus width. Live, lineage negative CD3+ T cells were subsequently gated and CD8+ T cells were identified within this fraction. (b) Antigen specific cells at Days 10, 30, and 148 are depicted using the sorting gate drawn on HLA-B7:RPIDDRFGL dextramer binding cells.

Fig. S6

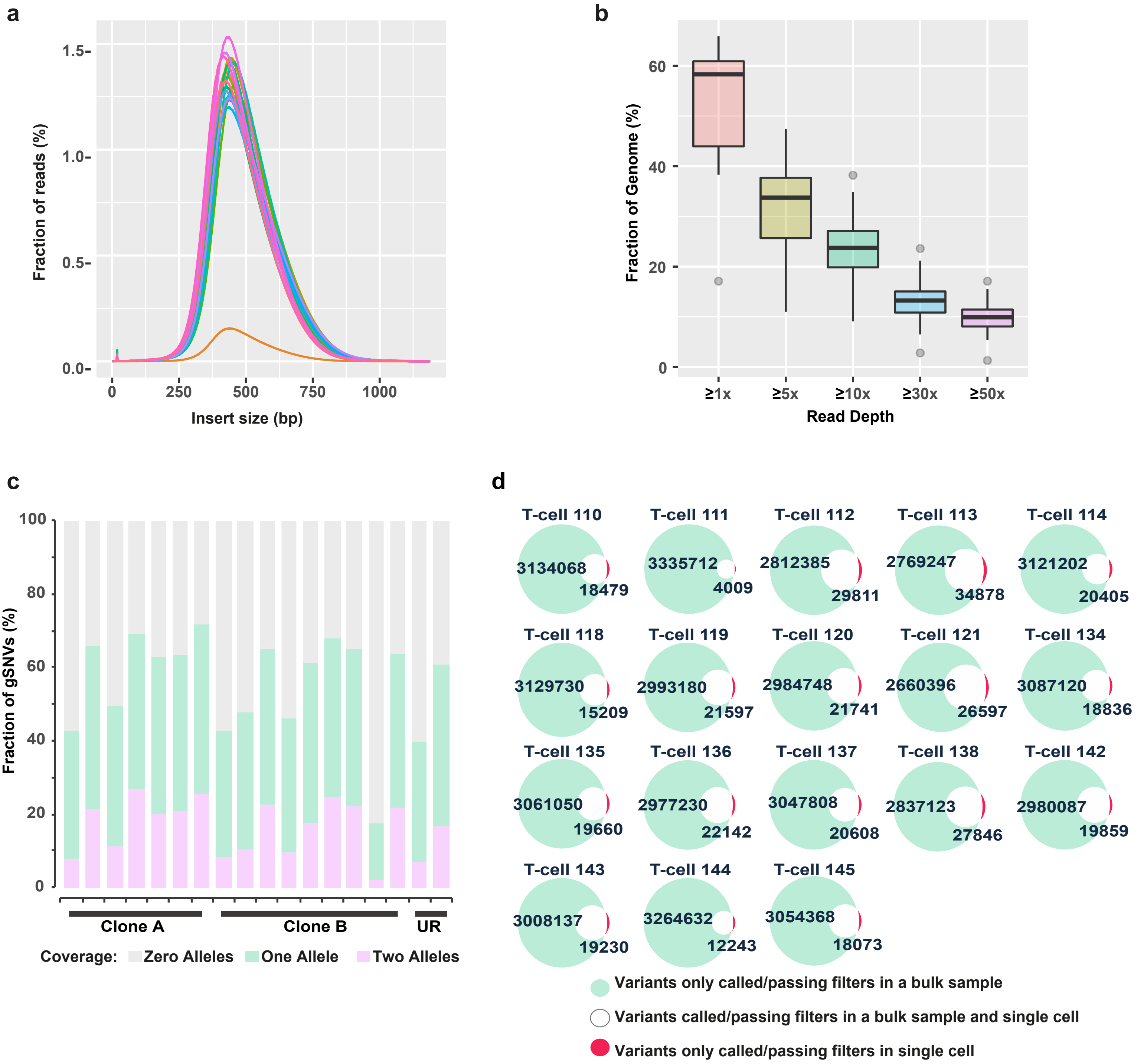

Fig. S6. Data quality statistics in MDA-amplified single T cells (a) Distribution of insert sizes in sequencing libraries from single T cells. (b) The fraction of the genome covered by at least 1, 5, 10, 30 and 50 reads per single T cell sample (c) Amplification efficiency in single T cells belonging to two clones (A and B) and unrelated cells (UR). Amplification efficiency in single T cells was estimated by analysis of read coverage in heterozygous gSNV sites, where reads originating from zero alleles represent locus dropout, reads originated from one represent allelic dropout, and reads originating from two alleles represent sites with full coverage (no allelic dropout). Only reads and bases with a mapping quality and base quality equal or greater than 20 were considered. (d) Overlap of variants called by Freebayes, in an unamplified bulk sample and single T cells. Only variant calls supported by at least 20 reads with a GQ score of at least 20 were considered.

Fig. S7

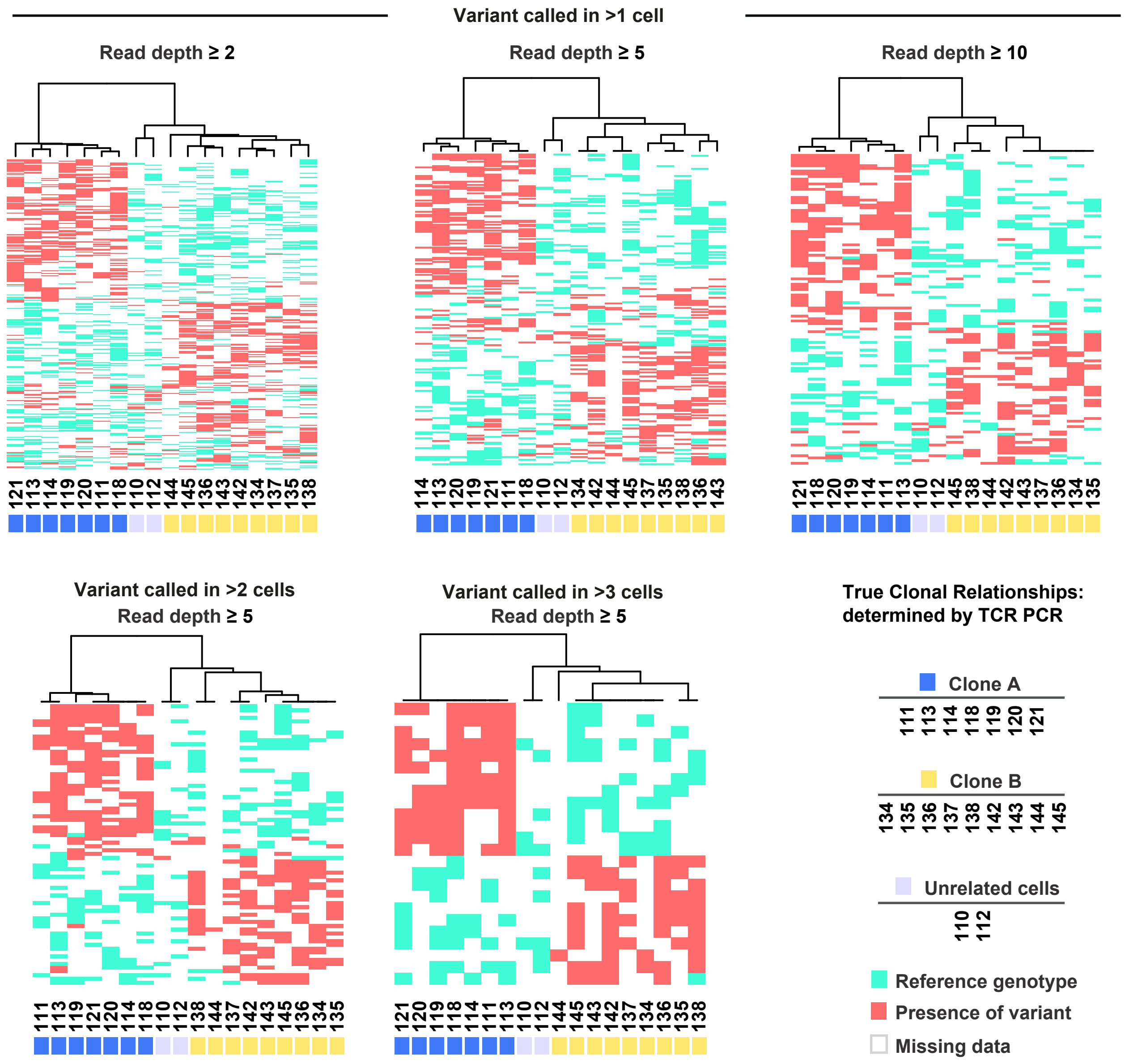

Fig. S7. Hierarchical clustering of single T cells using genotypes called by Conbase to define distances between single cells, using three different read depth filters and 3 different filters based on presence variant in multiple cells. Distances were defined as unknown if no shared sites were detected. For shared sites, the distance was decreased with -1 for each site where cells shared a mutation or increased with +1 if the genotypes of the cells differed. The distance matrix was then clustered using standard hclust with the distance 'ward.D2'.

Fig. S8

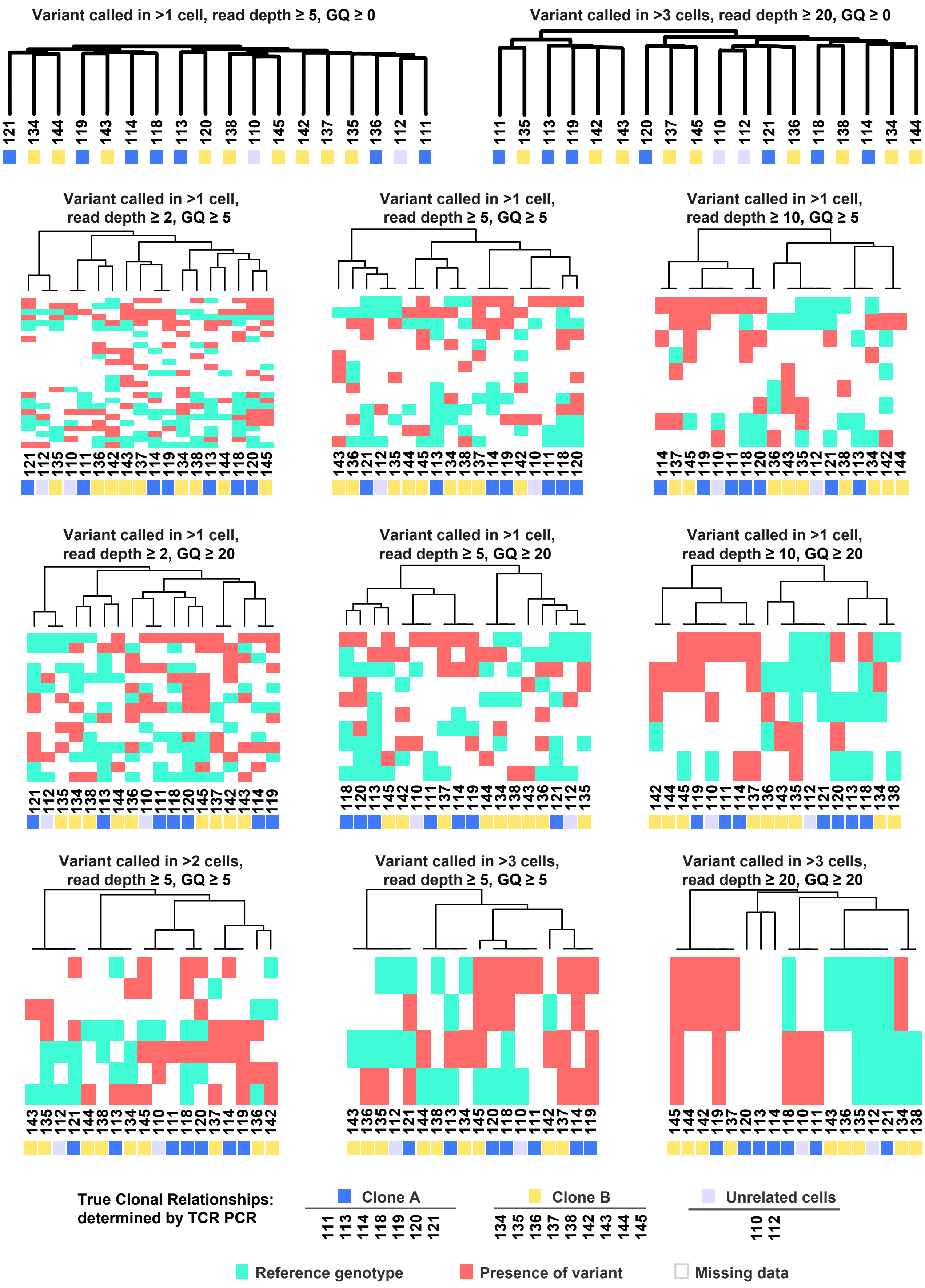

Fig. S8. Hierarchical clustering of single T cells using genotypes called by Monovar to define distances between single cells, using three different read depth filters and 3 different filters based on presence variant in multiple cells. Distances were defined as unknown if no shared sites were detected. For shared sites, the distance was decreased with -1 for each site where cells shared a mutation or increased with +1 if the genotypes of the cells differed. The distance matrix was then clustered using standard hclust with the distance 'ward.D2'.

Fig. S9

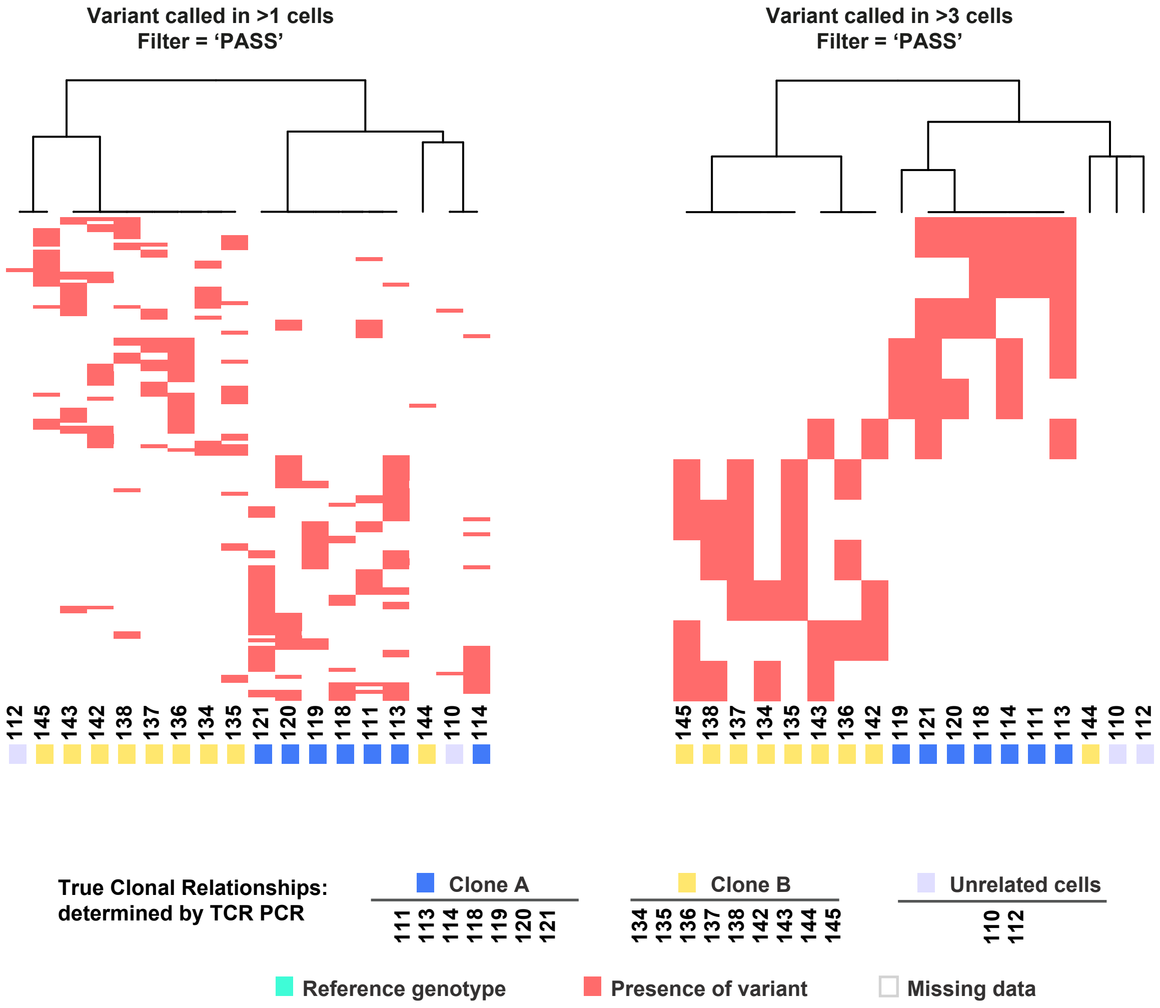

Fig. S9. Hierarchical clustering of single T cells using genotypes called by LiRA to define distances between single cells. Distances were defined as unknown if no shared sites were detected. For shared sites, the distance was decreased with -1 for each site where cells shared a mutation, otherwise the distance was defined as not available. The distance matrix was then clustered using standard hclust with the distance 'ward.D2'.
