# Supplementary material for: Conbase: a software for unsupervised discovery of clonal somatic mutations in single cells through read phasing
Source: Genome Biol. 2019 Apr 1;20:68. doi: 10.1186/s13059-019-1673-8 (PMC6444814; doi:10.1186/s13059-019-1673-8)
Supplement: Supplementary file 4 — Simulation model. A detailed description of the model used for generating the simulated data included in this study. (PDF 117 kb) [file 13059_2019_1673_MOESM4_ESM.pdf]

1 Notation

1.1 Loci and Cells

We will generate reads for a set L of loci in a set C of cells. A locus in L is defined as comprising two sites S and G, close enough to be covered by a (paired-end) read. The cells in C are assumed to form a related population.

1.2 States of G

For a locus $l \in L$, the genome states of G are referred to as $g1/g2$, where $g1$ is the nucleotide state on allele 1 and $g2$ is the state on allele 2. G will always be heterozygotic and, moreover, the assignment of $g1$ and $g2$ to maternal and paternal alleles are made such that $g1 = R$ and $g2 = A$, where R is the reference allele, and A is the alternative allele. This assignment is consistent within l and over C.

1.3 States of S

The genome states of S are similarly referred to as $s1/s2$, where $s1$ and $s2$ are located on alleles 1 and 2, respectively, as determined by the states of G above. The states of S are determined by the simulation: If S is a generated as a sSNV in the population, the state of S can be homozygotic or heterozygotic for individual cells, while if S is not an sSNV, all cells will be homozygotic for S. In the former case, all heterozygotic cells will will have the same of two mutually exclusive possible genotypes, (i) $s1 = R$ and $s2 = A$ or (ii) $s1 = A$ and $s2 = R$, where R is the reference allele found in bulk and A is a specified alternative state. For simplicity of explanation, we will w.l.o.g. in the following text assume case (i) when discussing a sSNV locus. For all homozygotic loci, we always have $s1 = s2 = R$. The described setup is consistent within l, but may, as indicated, vary over C.

1.4 Haplotypes and genotypes

The haplotype for each allele, with respect to S and G will be referred to as a tuple comprising the state of S and the state of G, in order, e.g., the haplotype AR for allele i means that $s_i = A$ and $g_i = R$.

Under our assumptions above, there are only two possible *genotypes*, i.e., pairs of haplotypes for a locus, either with a homozygote S: $\{RR, RA\}$, or with a heterozygote S: $\{RR, AA\}$.

For a cell c and a locus l, we will let $l(c)$ refer to the genotype of l in c and let $l_i(c)$ refer to the haplotype of allele i och l in c.

1.5 Population structure

To consistently model the distribution of mutated sSNVs among cells for all loci, we need to describe a population structure of the cells. A nice, general way to do this, allowing for any scenario we might want to use, is to define the relationship between cells as a tree, T, with internal nodes indexed, e.g., by numbers, and cells at the leaves. The tree needs not be perfectly binary, but will typically have polytomies – in a very simple scenario, this could be a tree with two internal nodes (clades) representing two clones of cells (plus a root node).

The internal node numbers can be viewed as indexing sets of related cells (clades). Let V be the set of numbers indexing internal vertices, excluding the root of T.

When we, for a given sSNV locus, want to simulate which cells should be heterozygotic for S, we simply draw a internal node number randomly from V and set all cells in the corresponding set/clade to be heterozygotic; all remaining cells are set to be homozygotic. In our simple two-clone tree example, this would correspond to making all cells in one clone heterozygotic and all cells in the other clone homozygotic.

Since we use the same tree for all loci, all sSNVs will be consistent with the population structure.

Notice that, by using a tree with a minimum clade size (e.g., no clade with less than m cells will have a number), we can avoid generating cases where the mutated state occurs only in a single (or few) cell(s), while retaining some biological realism.

1.6 Empirical Reads sets

For each locus, l, we will work with four sets of reads covering a locus with S and G; The reads are derived from bulk-DNAseq of two different cell populations, one of which one is heterozygous for S and the other is homozygous for S; both are heterozygous for G. The reads sets derived from each allele of the heterozygous population are called *hetRef* and *hetMut*, respectively. Similarly, the reads sets from the homozygous population are called *homRef* and *homMut*. We will, w.l.o.g., assume that the allele assignments and the G and S states are defined such that $hetRef = homMut = RR$.

1.7 Empirical Coverage distribution

We will make use of a empirically determined frequency distribution, d_{COV}, over reads coverage, i.e., the number of reads from each allele in a locus. The distribution d_{COV} is estimated over the reads for all loci and cells from a single-cell DNAseq experiment. This distribution is used i.i.d for all generated cells and loci.

2 Generation

The locus-specific read sets will be used to generate cell population scenarios comprising a locus l, which either is homozygotic for S in all cells or is a sSNV, i.e., some cells are homozygotic for S and other are heterozygotic, and a potential locus l', which either is homozygotic or heterozygotic for all cells; whether S is a sSNV is determined by a variable SNV. The locus l' will be used to simulate possible alignments errors (EAL) when predicting the genotype of l. In addition, we will simulate dropout of reads (DO) in l and l', while empirical distributions are used for allele-specific reads counts (C).

The strategy for generating reads is to use predefined probabilities of SNV, EAL, DO and the empirical distribution of C for l to step-by-step determine a count distribution f_R of number of reads to sample for cell $c \in C$, over the possible reads sets $R = \{l_1(c), l_2(c), l'_1(c), l'_2(c)\} \subseteq \{hetRef, hetMut, homRef, homMut\}$ for l in each cell c; notice that setting $f_R(h) = 0$ for a reads set h is equivalent to excluding sampling from h. We then sample reads following f_R to simulate sequencing reads mapping to l.

2.1 Generating SNV and determining R , or equivalently, assigning genotype to alleles of l and l'

We first determine SNV for a locus l, i.e., whether l will have an $sSNV$ in S ($SNV = 1$) or not ($SNV = 0$). This can be done using either of two strategies:

1. The locus l will have a sSNV at S ($SNV = 1$) with a pre-defined probability p_{SNV}, which is i.i.d. over all loci. This strategy is biologically more realistic.

2. We use a pre-defined f_{SNV}, the frequency of sSNV loci in L, and then sample random subset of size $k = f_{SNV}|L|$ loci from L and set $SNV = 1$ for these loci only. This allows more precise control of the observed frequency of sSNV in the generated data.

- If l has $SNV = 1$, then

    1. For all cells $c \in C$ initialize $l_i(c)$ and $l'_i(c)$ to homozygotic

$$\begin{aligned} l_1(c) &= homRef \\ l_2(c) &= homMut \\ l'_1(c) &= homRef \\ l'_2(c) &= homMut \end{aligned}$$

    2. Sample a cell subset V from the population tree
    3. For all cells $c \in V$, change $l(c)$ to heterozygotic

$$\begin{aligned} l_1(c) &= hetRef \\ l_2(c) &= hetMut \end{aligned}$$

- Otherwise ($SNV = 0$), for all cells $c \in C$, set $l(c)$ to homozygotic and $L'(c)$ to heterozygotic

$$\begin{aligned} l_1(c) &= homRef \\ l_2(c) &= homMut \\ l'_1(c) &= hetRef \\ l'_2(c) &= hetMut \end{aligned}$$

Notice that we have chosen to let the value of SNV also controls the read set assignment to l' in such a way as to create problems for variant calling.

2.2 Generating EAL

Similar to the case of SNV can determine whether a locus l will have an alignment error event (EAL) using two different strategies,

1. The locus l will have an EAL with a pre-defined probability p_{EAL}, which is i.i.d. over all loci.

2. We set f_{EAL}, the frequency of EAL loci in L, and then sample a random subset of size $k = f_{EAL}|L|$ loci from L and set $EAL = 1$ for these loci, only.

- If $EAL = 1$ the initial values of f_R for each $c \in C$ are set to:

$$\begin{aligned} f_R(l_1(c)) &= 1 && (1) \\ f_R(l_2(c)) &= 1 && (2) \\ f_R(l'_1(c)) &= 1 && (3) \\ f_R(l'_2(c)) &= 1. && (4) \end{aligned}$$

- Otherwise ($EAL = 0$), the initial values of f_R for each $c \in C$ are set to (i.e., excluding sampling from l'):

$$\begin{aligned} f_R(l_1(c)) &= 1 && (5) \\ f_R(l_2(c)) &= 1 && (6) \\ f_R(l'_1(c)) &= 0 && (7) \\ f_R(l'_2(c)) &= 0. && (8) \end{aligned}$$

Notice that presence of alignment errors is done on the population level.

2.3 Read dropouts

Presence of DO in a locus is modeled on a per-cell basis. $R_{DO} = \{l_1, l_2, l'_1, l'_2\}$.

Also, the presence of DO in c can be modeled in two ways:

1. For each cell $c \in C$ and for each allele $i \in R_{DO} = \{l_1, l_2, l'_1, l'_2\}$, set $f_R(i) = 0$ with a pre-defined probability p_{DO}, which is i.i.d. over all cells and alleles.

2. Using a pre-defined frequency of DO in Cf_{DO}, we sample a random subset, C_{DO}, of $k = f_{DO}|C|$ cells from C. For each $c \in C_{DO}$, we randomly sample an allele l_{DO} from $R_{DO} = \{l_1, l_2\}$ and set $f_R(l_{DO}) = 0$.

   Notice that we in this case model DO only on l; the reasoning for this is to simplify siulation while focusing on the problematic cases for variant calling.

2.4 Generating COV, the number of reads per allele

We sample the number of reads $COV(i)$ for each allele $i \in R$ i.i.d. from an empirical allele-specific coverage distribution d_{COV}. Typically, we set a minimum value for COV (e.g., 10) to avoid generating loci that will be filtered due to low coverage.

We then set

$$\begin{aligned} f_R(l_1(c)) &= COV(l_1) f_R(l_1(c)) \\ f_R(l_2(c)) &= COV(l_2) f_R(l_2(c)) \\ f_R(l'_1(c)) &= COV(l'_1) f_R(l'_1(c)) \\ f_R(l'_2(c)) &= COV(l'_2) f_R(l'_2(c)). \end{aligned}$$

Notice that, if the 'input' $f_R(i) = 0$ *(to right of the equal sign), then it is guaranteed that also the 'output'* $f_R(i) = 0$ *(to the left of the equal sign).*

2.5 Sample reads from f_R

For cell c and locus l, we will now create $r_{l,c}$, the set of reads mapping to l in c.

1. Initiate $r_{l,c} = \emptyset$.
2. for $i \in \{l_1(c), l_2(c), l'_1(c), l'_2(c)\}$
   (a) Repeat the following $f_R(i)$ times
      i. Draw a read, r, from i and add it to $r_{l,c}$, i.e., $r_{l,c} = r_{l,c} \cup \{r\}$.

For each $c \in C$, the set of reads, $\{r_{*,c}$, is saved to a separate bam-file.
This concludes the description of the generative model and algorithm.
