# Supplementary material for: Conbase: a software for unsupervised discovery of clonal somatic mutations in single cells through read phasing
Source: Genome Biol. 2019 Apr 1;20:68. doi: 10.1186/s13059-019-1673-8 (PMC6444814; doi:10.1186/s13059-019-1673-8)
Supplement: Supplementary file 7 — Review history. (DOCX 87 kb) [file 13059_2019_1673_MOESM7_ESM.docx]

Review History

**First round of review**

**Reviewer 1**

**Are the methods appropriate to the aims of the study, are they well described, and are necessary controls included?**
yes

**Are the conclusions adequately supported by the data shown?**

Yes

**Are sufficient details provided to allow replication and comparison with related analyses that may have been performed?**

Yes

**Does the work represent a significant advance over previously published studies? Please explain.**

yes, as demonstrated by direct comparison with available related software

**Is the paper of broad interest to others in the field, or of outstanding interest to a broad audience of biologists?**

No

**Comments to author:**

In this manuscript the authors developed a software to call somatic mutations from single cell genome sequencing data. The main strength of their method is that it considers the compatibility among different cells of the same individual and uses the information of haplotype. The manuscript can be read as two parts: the first part studies simulated data, and the second part studies real data that are generated with design by the authors themselves. While the second part looks nice, the first part has apparent flaws. Below are the specific comments:

1.	As for the basic logic associated with Fig.1, a previous paper has already discussed (Cong Ji, et al, A Simple Strategy for Reducing False Negatives in Calling Variants from Single-Cell Sequencing Data, PLoS One, 2015).

2.	It seems that the false negatives and false positives are separately addressed using different simulated data, which is not the correct way of evaluating false discoveries.

3.	In addition, results in Fig. 2b, 2c, and 2e are simply too good to be true. It reflects the oversimplification of the simulation and thus would mislead the readers. The authors should do the simulation based on a real human genome. This way, the complexity of the genome as well as the density of human SNPs can be better modeled.

4.	One of the major sources of false positives is actually allele "drop-out" of the bulk sample (i.e., low sequencing coverage of some regions). This deserves a special consideration.

5.	When the read data is considered, the distribution plausibility of the called mutations tests mainly the false positives. How is the false negatives?

**Reviewer 2**

**Are the methods appropriate to the aims of the study, are they well described, and are necessary controls included?**
yes

**Are the conclusions adequately supported by the data shown?**

Yes

**Are sufficient details provided to allow replication and comparison with related analyses that may have been performed?**

Yes

**Does the work represent a significant advance over previously published studies? Please explain.**

Needs further evaluation by the authors.

**Is the paper of broad interest to others in the field, or of outstanding interest to a broad audience of biologists?**

Yes. Although a few single-cell sSNV callers or calling strategies have been published, I think it is still an important area of method development, because accurate variant calling from single cells is at the moment one of the major limiting factors for downstream applications of single-cell genomes, e.g., PGD, cancer, aging, and diseases associated with genome instability.

**Comments to author:**

In the manuscript, entitled "Conbase: a software for unsupervised discovery of clonal somatic mutations in single cells through read phasing", Hard et al., reported a variant caller for detecting somatic single nucleotide variations (sSNVs) from single cells. Although a few single-cell sSNV callers or calling strategies have been published, I think it is still an important area of method development, because accurate variant calling from single cells is one of the major limiting factors for downstream applications of single-cell genomes, e.g., PGD, cancer, aging, and diseases associated with genome instability. The single-cell variant calling was challenged by substantial additional errors and bias to conventional bulk sequencing, which Hard et al try to resolve using "read phasing". However, I have the following concerns.

Major

1.	What are the differences between Conbase and the "linked-read" calling published by Peter J Park group (https://www.ncbi.nlm.nih.gov/pubmed/29217584; https://www.biorxiv.org/content/early/2017/10/30/211169) at Harvard? The two methods, seem to me, are based on the same concept - when a candidate SNV and a SNP always are found always on same reads, the candidate SNV is inferred as a true SNV, otherwise a false one.

2.	Following the first question, in addition to comparing to Monovar, I suggest the authors compare Conbase to the "linked-read" calling.

3.	Another related comment, the sensitivity of variant calling developed under this concept may be low, although it is of high specificity, because there are only ~5 million SNPs in the human genome of 3GB. So what is the sensitivity of Conbase?

4.	Although the authors argued that Conbase can be applied in extreme situations, e.g., high dropout or error rate, which often/occasionally happens, these extreme situation can be minimized by optimizing experimental procedures. There are already several publish data sets available, with much less bias than the authors' experiment, e.g., Lodato Science 2015, Dong Nat Methods 2017, Chen Science 2017. Therefore, I think it is important that the authors also evaluate Conbase when single-cell amplifications are well carried out. The authors may choose randomly 1% germline heterozygous SNPs, regard them as sSNVs and test Conbase on these "sSNVs". The authors can compare Conbase to Monovar, "linked-read" as well as SCcaller, which as pointed out in the manuscript is feasible to relatively well amplified cells.

5.	Related to the above suggestion and just a comment, it is strange that in the authors experiment, the amplification suffered from such severe dropout. Therefore, published data, I think, is a better resource for evaluation.

6.	Performance evaluation in the manuscript, in my opinion, can be carried out in a more fair manner. When reporting specificity, sensitivity in the same evaluation should also be reported, and vise versa. For example, in Figure 3, although most of variants detect by Conbase are plausible (~90%, the right panel), Conbase may missed 99% of the total plausible mutations (the left panel, detected by Monovar)!

7.	Results in Figure 4 are very impressive, but the authors should include the "linked-read" analysis in the comparison.

Minor

1.	In the title, what does "unsupervised" refer to?

2.	Line 388 "Monovar is the most widely used variant caller for single cell DNA sequencing data exhibiting allelic dropout and amplification bias." This claim may not be true. All the tools, as far as I know, have been used only once (when published) at the moment, because single-cell whole-genome sequencing is still at the moment not easy to carry out.

**Authors Response**

**Point-by-point responses to the reviewers’ comments:**

Reviewer #1: In this manuscript the authors developed a software to call somatic mutations from single cell genome sequencing data. The main strength of their method is that it considers the compatibility among different cells of the same individual and uses the information of haplotype. The manuscript can be read as two parts: the first part studies simulated data, and the second part studies real data that are generated with design by the authors themselves. While the second part looks nice, the first part has apparent flaws. Below are the specific comments:

*We are happy that this reviewer acknowledges the experimental design and analysis of the real world datasets generated by our group. The points raised by this reviewer regarding the first part including simulated data are well taken and we have addressed these issues in the revised manuscript. Please see the specific responses below.*

1. As for the basic logic associated with Fig.1, a previous paper has already discussed

(Cong Ji, et al, A Simple Strategy for Reducing False Negatives in Calling Variants from

Single-Cell Sequencing Data, PLoS One, 2015).

*We agree that the strategy described by Cong Ji et al to reduce false negative genotypes, follows the same basic logic as used in Conbase. However, in addition to using heterozygous germline single nucleotide variants to exclude incorrectly predicted homozygous genotypes, Conbase utilizes the phasing information across a population of cells to simultaneously exclude false positive variant calls, represented by conflicting genotypes as indicated in Figure 1. To our knowledge, Conbase is the only available tool that enables exclusion of incorrectly predicted homozygous genotypes and false positive variant calls by taking advantage of phasing using heterozygous germline single nucleotide variants. In order to direct readers to a more detailed overview explaining how phasing information is used in Conbase, we have revised the presentation of this in the Supplementary Material. Supplementary Figure 1 (previously Supplementary Figure 8) outlines a schematic overview of the major data processing functions of the algorithm, and we have included references to both Figure 1 and Supplementary Figure 1 on the same line in the main text of the manuscript.*

2. It seems that the false negatives and false positives are separately addressed using different simulated data, which is not the correct way of evaluating false discoveries.

*We thank this reviewer for pointing out that it is not optimal in this case to address false negatives and false positives in separate datasets to evaluate false discoveries. In the revised manuscript, we have substantially restructured our simulation strategy and its description, including simultaneous testing of false positives and false negatives as requested by this reviewer.*

3.	In addition, results in Fig. 2b, 2c, and 2e are simply too good to be true. It reflects the oversimplification of the simulation and thus would mislead the readers. The authors should do the simulation based on a real human genome. This way, the complexity of the genome as well as the density of human SNPs can be better modeled.

*We agree with this reviewer that the biological realism of the model used to generate data is crucial for the relevance of the simulation study. In the revised manuscript, we have therefore substantially restructured our simulation analysis strategy. This was done in order to allow estimation of relevant statistics and to better reflect how the complexity of the human genome affects variant calling, as requested by this reviewer. We focus on the effect of ambiguous read mapping which represents the major source of false positives in variant calling, corresponding to 1 error in 10kb-15kb (Li Bioinformatics 2014). In the revised manuscript, we now clarify that such alignment artifacts are the most likely source of errors when calling clonal somatic mutations also in single cell data. In our new simulation strategy, we evaluate variant calling performance at increasing probability for alignment artifacts to occur. In this setting, individual reads represent molecules derived from multiple locations in the genome with high sequence similarity, including paralogs, structural variation or low-complexity genomic DNA. To construct a model that is relevant for single cell variant calling, we simultaneously investigate the effects of features that are specific for single cell data, including allelic dropout and amplification bias. To address the problem of biological reality, we base our simulation model on loci, reads and coverage from real data representing bulk and single cells. We perform two simulation analyses, one aimed at testing the identification of somatic variants in a population of cells, and the second aimed at testing classification of the genotypes of individual cells in somatic variant loci.*

*An important contribution of the simulation analyses is that it allows us to evaluate sensitivity (TP/(TP+FN)), specificity (TN/(TN+FP)) and false discovery rate (FP/(FP+TP)) of the tested methods, and therefore add a substantial amount of additional information. To better demonstrate the novelty of Conbase over existing tools for variant calling in single cells, we have included comparative analyses with two additional methods in the revised manuscript. On simulated data, we have evaluated the sensitivity, specificity and false discovery rate of Conbase, Monovar, SCcaller (Dong et al Nature Methods 2017) and Linked Read Analysis (LiRA) (Bohrson et al bioRxiv 2017). In summary, Conbase, Monovar and LiRA performs comparably in terms of the sensitivity to detect simulated somatic variant sites, and achieves considerably better sensitivity over SCcaller (Figure 2, Supplementary Figure 3). Notably, Conbase achieves substantially better specificity as well as false discovery rate than Monovar, LiRA and SCcaller, indicating superior ability to distinguish artifacts (Figure 2, Supplementary Figure 3).*

*A limitation of our simulations is that we are confined to study distinct genomic windows, which may not capture the full complexity of alignment artifacts expected in real data. In the revised manuscript we discuss this issue. Simulating alignment artifacts is challenging, given that the distribution and frequency of structural variation and low complexity sequences in the human genome is not fully characterized. This is in part due to the difficulty of quantifying these from short read sequencing data (Huddleston et al, Genome Research, 2017). Despite this limitation of our simulation study, we believe that we now provide a comprehensive description of our analysis of simulated data, and that it provides biologically plausible simulation results. In the revised manuscript, the results from the simulation experiments are summarized in the introduction and described in the results section, including Figure 2 and Supplementary Figure 3. The simulation model is described in the methods section, as well as a more formal and detailed description in Supplementary Text 1.*

4.	One of the major sources of false positives is actually allele "drop-out" of the bulk sample (i.e., low sequencing coverage of some regions). This deserves a special consideration.

*This reviewer is correct in that germline variants may incorrectly be interpreted as somatic variants due to the absence of variant supporting reads in a bulk sample. We thank the reviewer for pointing this out, and we address this issue in the revised manuscript. Additionally, observing single cells with unmutated genotypes increases the confidence that a site represents a somatic variant in the population of cells, and not a germline variant that would be present in all cells. When evaluating the results obtained from Conbase and Monovar on real data, we specifically analyze sites in which at least one sample is predicted to be unmutated. The increased confidence that called somatic variants are not germline variants, on the basis of observing individual cells with unmutated genotypes, depends on the ability of a method to correctly predict unmutated genotypes. In our simulation analyses, we see that Conbase performs very well in correctly predicting unmutated genotypes in single cell data. In contrast, as the rate of dropout increases, so does the fraction of incorrectly predicted unmutated genotypes in Monovar output. Among the tested methods, only Conbase and Monovar enable predictions of unmutated states of cells, whereas SCcaller and LiRA does not predict unmutated genotypes. As such, this feature provides an important distinction between the variant callers. This has been clarified in the revised manuscript. Additionally, we discuss the advantage of requiring to observe single cells with unmutated genotypes, to obtain increased confidence that called somatic variants are not germline variants.*

5.	When the read data is considered, the distribution plausibility of the called mutations tests mainly the false positives. How is the false negatives?

*A target application for our tool is to perform lineage tracing of human cells, where the distribution of called somatic variation is used as input data for phylogenetic analysis and reconstruction of phylogenetic trees reflecting cell lineage relationships. In this setting, it is of utmost importance to minimize the number of falsely predicted somatic variants, as these introduce noise and misleading signals, severely reducing the power of phylogenetic analyses. Li (Bioinformatics 2014) showed that errors in variant calling in whole genome sequencing data is very abundant (1 error per 10kb – 15 kb), primarily resulting from alignment errors and the limitation of using a reference genome with respect to the genome of the donor. In addition, errors introduced during the whole genome amplification procedure may produce similar problems, but appear to be more rare (Bourcy et al Plos One 2014). Given the high number of false signals, and that the signal from true somatic variation is very low (Yu et al Nature 2017), exclusion of false positives is crucial in order to enable lineage tracing at the single cell level, whereas the effect of false negatives is less of a problem in phylogenetic analysis. Nevertheless, it is very valuable to identify false negatives.*

*Methods that rely on read phasing, including Conbase and LiRA, will fail to call somatic variants which are not present in the same read or read pair as at least one heterozygous germline single nucleotide variant. The prevalence of this type of false negatives can be directly estimated from the fraction of the genome that can be phased, which depend on the frequency and distribution of heterozygous germline single nucleotide variants, as well as the insert size of the sequencing libraries. For an insert size of 600bp, approximately 50% of the genome is phasable. In the revised manuscript we have included a graph representing the fraction of the genome in which variant calling is performed by the tested methods (Figure 1b).*

*To evaluate the prevalence of false negatives in a controlled setting, we restructured our simulation strategy. In our new simulation strategy, we perform two experiments addressing two types of false negatives. In the first experiment we focus on the detection of loci, in which a somatic variant is observed in the population of cells. In this setting, a false negative corresponds to the failure to detect a somatic variant locus. Here, we follow the common consensus that loci displaying alignment errors, are not callable and would be classified as negatives. Given phasable somatic variants, we perform simulation studies designed to quantify the effect of alignment errors and allelic dropout, reported by false positive rates, specificity and sensitivity statistics. Our results show that Conbase, Monovar and LiRA all performs well in terms of sensitivity whereas SCcaller performs worse. In the second experiment, we evaluate the performance of the tested methods to correctly predict the genotypes of individual cells in generated somatic variant loci. Here, we test the impact of allelic dropout and investigate six different statistics. These include true correctly predicted heterozygous genotypes, incorrectly predicted heterozygous genotypes, correctly predicted homozygous genotypes, incorrectly predicted homozygous genotypes, no prediction when the true genotype is heterozygous, no prediction when the true genotype is homozygous. In Conbase output, there are no incorrectly predicted homozygous genotypes. In Monovar output, the fraction of incorrectly predicted homozygous genotypes increases with dropout probability. Monovar correctly predicts homozygous genotypes to a higher extent as compared to Conbase. There are no incorrectly or correctly predicted homozygous genotypes in output from SCcaller or LiRA. Among the tested methods, Monovar makes no prediction the least number of times, followed by Conbase. SCcaller make no prediction the highest number of times.*

Reviewer #2: In the manuscript, entitled "Conbase: a software for unsupervised discovery of clonal somatic mutations in single cells through read phasing", Hard et al., reported a variant caller for detecting somatic single nucleotide variations (sSNVs) from single cells. Although a few single-cell sSNV callers or calling strategies have been published, I think it is still an important area of method development, because accurate variant calling from single cells is one of the major limiting factors for downstream applications of single-cell genomes, e.g., PGD, cancer, aging, and diseases associated with genome instability. The single-cell variant calling was challenged by substantial additional errors and bias to conventional bulk sequencing, which Hard et al try to resolve using "read phasing". However, I have the following concerns.

Major

1. What are the differences between Conbase and the "linked-read" calling published by

Peter J Park group

(https://www.ncbi.nlm.nih.gov/pubmed/29217584;https://www.biorxiv.org/content/early/201

7/10/30/211169) at Harvard? The two methods, seem to me, are based on the same concept - when a candidate SNV and a SNP always are found always on same reads, the candidate SNV is inferred as a true SNV, otherwise a false one.

*There are two major differences between Conbase and Linked Read Analysis (LiRA) (1) Conbase performs joint variant calling, in which read observations are considered across the single cell population. In contrast, LiRA performs variant calling individually per sample. Through joint variant calling, information is gained on the population level and is used to improve predictions for individual cells. This allows for increased confidence in the called somatic variants and genotypes can be predicted at low read depth (down to 2x).*

*(2) Conbase predicts both the presence as well as the absence of mutations, whereas LiRA predicts only the presence of mutations. Identifying cells with unmutated genotypes within the population, is required for distinction between clonal mutations and subclonal mutations. In addition, predicting the absence of mutations in single cells is required to distinguish somatic variation from germline variation. This is because dropout is expected also in bulk data, as pointed out by reviewer 1. Importantly, information about mutated and unmutated states of cells is crucial for many downstream applications for single cells genomics, including phylogenetics and lineage tracing at the single cell level, which are target applications for Conbase.*

*We discuss these differences between Conbase and LiRA in the revised manuscript.*

2. Following the first question, in addition to comparing to Monovar, I suggest the authors compare Conbase to the "linked-read" calling.

*In the revised manuscript we have included comparative analyses with LiRA in simulated and real data, as requested by this reviewer. In our response to comment 4 by this reviewer, we summarize the results from the comparative analyses on simulated data. In our response to comment 7 by this reviewer, we summarize the results from the comparative analyses on real data.*

3. Another related comment, the sensitivity of variant calling developed under this concept may be low, although it is of high specificity, because there are only ~5 million SNPs in the human genome of 3GB. So what is the sensitivity of Conbase?

*We apologize for the lack of clarity regarding the limitation of using phasing during variant calling. The prevalence of false negative variant sites can be directly estimated from the fraction of the genome that can be phased, which depend on the frequency and distribution of heterozygous germline SNVs, as well as insert size of sequencing libraries. Based on the observed frequency and distribution of germline SNVs in our two donors, we have computed the fraction of bases that can be phased at different sequencing library insert sizes (Supplementary Figure 9). With an average insert size of 600 bp, ~50% of the genome can be phased. In the revised version of the manuscript we have included a graph representing the fraction of the genome in which variant calling can be performed by the tested methods (Figure 1b). We have also clarified this issue in the main text.*

4. Although the authors argued that Conbase can be applied in extreme situations, e.g., high dropout or error rate, which often/occasionally happens, these extreme situation can be minimized by optimizing experimental procedures. There are already several publish data sets available, with much less bias than the authors' experiment, e.g., Lodato Science 2015, Dong Nat Methods 2017, Chen Science 2017. Therefore, I think it is important that the authors also evaluate Conbase when single-cell amplifications are well carried out. The authors may choose randomly 1% germline heterozygous SNPs, regard them as sSNVs and test Conbase on these "sSNVs". The authors can compare Conbase to Monovar, "linked-read" as well as SCcaller, which as pointed out in the manuscript is feasible to relatively well amplified cells.

*Previously published data indeed demonstrate high genome coverage and low rates of allelic dropout, including the work mentioned above. It is important to note however, that many published methods for single cell genomics have been benchmarked using cell lines and rapidly dividing cells, which may allow optimal data quality. Many cell types for which single cell genomics would be useful are situated in discrete tissue compartments, and in order to retain the spatial information about cells it is often necessary to perform isolations using laser capture microdissection, resulting in additional loss of genomic DNA upstream of amplification procedures. In our opinion, robust variant calling that is not dependent on specific data quality thresholds is an advantage, in particular for clinical applications for single cell genomics where donor material is scarce and DNA integrity may be compromised.*

*We would also like to point out that a large variance in terms of whole genome amplification efficiency has been described in the literature, including the studies referenced above by the reviewer. While the data included in Lodato et al. (Science 2015) display low rates of allelic dropout, additional data generated by the same lab using the same in-house multiple displacement amplification (MDA) protocols and included in Lodato et al. (Science 2018; Supplementary Figure 2G), display allelic dropout rates which are comparable to our dataset. The increased rate of allelic dropout observed in Lodato et al. (Science 2018), is supported by Zong et al. (Science 2012), reporting ~65% allelic dropout rate in MDA data. Also for multiple annealing and looping based amplification (MALBAC), amplification efficiency appear to vary, even in the hands of the developers of the method. In the original paper describing MALBAC, Zong et al. Science 2012, demonstrate low allelic dropout rates. However, in the study conducted by Chen et al. (Science 2017) from the same lab, a 40-50% allelic dropout rate was reported in MALBAC data (Supplementary Table 1). The reported variance in amplification efficiency may be affected by uncharacterized technical, as well as biological aspects of single cell data preparations. These may include batch effects of whole genome amplification enzymes or properties of the nucleic acids to be isolated and amplified, such as cell type specific chromatin structures and even post mortem intervals in the case of tissues from deceased donors. In this regard, Lodato et al 2015 (and 2018) isolated DNA from processed neuronal nuclei which may aid in DNA dissociation early in the amplification protocol and which is not possible for our clonal T cell analysis which relies on sorting cells based on proteins on the cell surface.*

*We agree with the reviewer that germline variation is a useful source of data for simulating somatic variation. In the revised manuscript, we have performed extensive analyses of simulated data, in which germline variation is used to model somatic variation, as suggested by this reviewer. Here we generated a statistical model, which allows us to evaluate sensitivity and specificity of variant calling and genotyping, in the presence of varying degrees of errors and dropout. To specifically evaluate Conbase also at low rates of dropout, as requested by this reviewer, we simulated dropout rates ranging from zero percent up to 90%. We perform two variants with respect to coverage uniformity. In one variant we use a flat rate of amplification bias, where each site is covered by 30 reads from each allele. In a second variant we use the coverage distribution observed in our single cell data from the CD8+ T cell donor. In the revised manuscript, we have also included comparative analyses with SCcaller and LiRA, as requested by this reviewer. In summary, the results show that Conbase, Monovar and LiRA performs comparably in terms of sensitivity to detect sSNV sites in a population of cells, whereas SCcaller performs worse. The sensitivity of SCcaller is improved in simulated data exhibiting no amplification bias, albeit lower than the other tested methods. Conbase outperforms the other tested methods in terms of specificity and false discovery rate. Conbase, Monovar and LiRA performs comparably in terms of correctly predicting heterozygous genotypes in individual samples, whereas SCcaller predicts fewer heterozygous genotypes in data with no amplification bias as well as in data exhibiting amplification bias. A major distinction between the tested methods is that only Conbase and Monovar predict both mutated and unmutated states of cells, whereas SCcaller and LiRA only predicts the presence of variants. A target application for our tool is tracing cell lineage, where the distribution of somatic variants among cells in a population is used as data in a phylogenetic analysis to construction trees reflecting their developmental relationship. Phylogenetic analysis requires input data including mutated and unmutated states of cells. As such, the output provided by SCcaller and LiRA is not sufficient to perform downstream phylogenetic analysis at the single cell level.*

*Taken together, in the revised manuscript we have evaluated variant calling performance in simulated data by using germline variants with known genotypes, as suggested by this reviewer. In our new simulation strategy, we apply increasing rates of allelic dropout and amplification bias in order to evaluate variant calling performance also in data exhibiting very low rates of allelic dropout, as requested by this reviewer. To demonstrate the novelty of Conbase over existing methods, we have included comparative analyses with two additional methods, SCcaller and LiRA, as requested by this reviewer. Overall, Conbase performs best of the tested methods, with regards to specificity and false discovery rate. Given phased read data, Conbase performs comparably to Monovar and LiRA in terms of sensitivity, while SCcaller performs worse.*

5. Related to the above suggestion and just a comment, it is strange that in the authors experiment, the amplification suffered from such severe dropout. Therefore, published data, I think, is a better resource for evaluation.

*We appreciate the suggestion by this reviewer to evaluate Conbase also on additional datasets. While we are familiar with work mentioned above (Lodato Science 2015, Dong Nat Methods 2017, Chen Science 2017), we do not agree that these datasets would enable us to comprehensively compare the performance of single cell variant callers to detect true clonal somatic variation. The dataset described in Lodato et al. Science 2015 contains single cells from the same individual, and the dataset described in Chen et al. Science 2017 contains single cells from the same cell line. However, neither of these datasets include known clonal relationships. In fact, a major conclusion from Lodato et al. is that the majority of identified mutations are only found in one cell making validation nearly impossible without access to more information. That is precisely why we took extra effort in our study to generate a dataset that could specifically address our questions. Only Dong et al (Nature Methods 2017) have generated a dataset where the clonal relationships are partially known. This dataset, obtained from cultured fibroblasts, contains only two single cells belonging to the same clone, whereas the phylogenetic distance to remaining single cells and between the remaining single cells is unknown. The small number of samples with known phylogenetic relationships in the dataset generated by Dong et al would severely limit our ability to assess the performance of the tested methods to call true clonal somatic variation in real world data.*

*Given the lack of publicly available datasets including clonal populations of single cells with multiple replicates, we instead simulated data according to the suggestion by this reviewer to use germline variants and regard these as somatic variants. The advantage of using simulated data generated from a statistical model is that we control the important parameters, enabling us to analyze data exhibiting a range of dropout rates. In the revised manuscript, we present the results from the simulation analyses demonstrating a superior performance of Conbase over the tested methods, in terms of specificity and false discovery rate.*

6. Performance evaluation in the manuscript, in my opinion, can be carried out in a more fair manner. When reporting specificity, sensitivity in the same evaluation should also be reported, and vise versa. For example, in Figure 3, although most of variants detect by Conbase are plausible (~90%, the right panel), Conbase may missed 99% of the total plausible mutations (the left panel, detected by Monovar)!

*This reviewer is correct in that Monovar holds an advantage over Conbase (and LiRA), because Monovar does not rely on read phasing. As such, Monovar can call variants in all genomic regions covered by reads, whereas Conbase and LiRA are restricted to bases in proximity to germline SNVs. The fraction of the genome which are present in the same read or read pair as at least one germline SNV corresponds to ~50% if the average insert size of sequencing libraries is 600 bp. To emphasize this advantage of Monovar, we have included a graph representing the genomic fraction in which variant calling is performed by Conbase, Monovar and LiRA (Figure 1b). However, Conbase and LiRA holds an advantage over Monovar in that these methods enable exclusion of false positive variant calls by the use of read phasing, while Monovar solely rely on base observations in individual positions. As we discuss in the manuscript, it appears reasonable to assume that false positive variant calls observed in multiple single cells are primarily result from alignment artifacts rather than amplification errors. This is because errors associated with read alignment are systematic and very abundant (1 error per 10kb – 15kb) (Li Bioinformatics 2014), while there is a low likelihood to acquire the exact same amplification error in multiple whole genome amplification reactions given a per cycle per base amplification error rate of 1 error per 105 -106 bases in MDA and 3 errors per 104 bases in MALBAC (de Bourcy et al Plos One 2014). While it is a common consensus that sites where alignment errors occur should be discarded*

*(Li Bioinformatics 2014, Zafar et al Nature Methods 2016, Yu et al Nature 2017), it can be extremely difficult to detect their presence just based on the base observations in individual positions of a given cell. However, alignment errors (and amplification errors) result in discordant alleles, and can thus more effectively be filtered out as false positive variant calls by the use of read phasing. By the use of single cell data representing clonal populations with known phylogenetic relationships, it is possible to compute the fraction of sites exhibiting biologically plausible and implausible distributions of genotypes. The distribution of genotypes can in turn, be used as a proxy allowing estimation of false discovery rate in real data where the true genotypes are unknown. Sites displaying biologically implausible genotype distributions are with high probability false positive variant calls, under the assumption that the probability of the same mutation occurring twice during evolution is extremely low (Kimura M. Genetics 1969). It is important to note however, that sites displaying biologically plausible genotype distributions can occur by chance due to missing data in the cell population. To test how the ratio between biologically plausible and implausible genotype distributions observed in Monovar output (Figure 3, left panel) relates to the ratio between biologically plausible and implausible genotype distribution that would occur by chance, we randomized the clonal relationships of the samples and computed the distributions of genotypes in the clones. In this experiment we repeated the randomization 10 times and calculated an average of observed fractions of sites displaying biologically plausible and implausible distributions of genotypes. The result demonstrates that the ratio between biologically plausible and implausible genotype distributions observed in Monovar output (right panel below, Figure 3a in the manuscript), is comparable to the ratio between biologically plausible and implausible genotype distributions occurring by chance (left panel below). This finding suggests that sites displaying biologically plausible genotype distributions in Monovar output may not all represent true somatic variation, and include artifacts that by chance display distributions of genotypes that are biologically plausible.*

7. Results in Figure 4 are very impressive, but the authors should include the "linked-read" analysis in the comparison.

*We were happy that this reviewer share our excitement over our results. We have included a comparative analysis with LiRA, as requested by this reviewer. The results demonstrate that Conbase and LiRA performs comparably in terms of enabling separation of clonal cell populations by hierarchical clustering. However, Conbase achieves more robust results than LiRA by identifying a higher number of clonal somatic variant sites. Moreover, Conbase predicts both mutated and unmutated states of cells. In contrast, LiRA only predicts the presence of variants.*

Minor

1. In the title, what does "unsupervised" refer to?

*Unsupervised refers to that no prior information about clonal relationships is provided as input to the software. This has been clarified in the revised manuscript.*

2. Line 388 "Monovar is the most widely used variant caller for single cell DNA sequencing data exhibiting allelic dropout and amplification bias." This claim may not be true. All the tools, as far as I know, have been used only once (when published) at the moment, because single-cell whole-genome sequencing is still at the moment not easy to carry out.

*We thank the reviewer for pointing this out and we have removed this sentence accordingly. We appreciate that this reviewer acknowledges the complexity of single cell whole genome sequencing.*

**Second round of review**

**Reviewer 1**

The manuscript has been extensively revised. I have no further comments.

**Reviewer 2**

In the revised manuscript, the authors included the comparison with LiRA and SCcaller, and changed the strategy of generating simulated dataset for testing purpose. However, as I pointed out previously, simulated dataset may be problematic, and reviewer 1 was in agreement with me and suggested that simulated dataset may oversimplify real situation. This problem can be solved by taking my suggestion to use real dataset publicly available (e.g., Lodato etal Science 2015, Dong et al Nat Methods 2017, Chen et al Science 2017). However, the authors disagreed and did not perform testing using public dataset. Below, I list the evidence that the current simulation is still problematic or misleading, which are the reasons that testing on real datasets publicly available (accepted) are necessary.

1. Sensitivities of SCcaller and Monovar estimated using the simulated dataset (Fig. 2A) contradicts to those estimated using real dataset (fig. 2c from Dong X et al. Nat Methods 2017).

2. Results in Fig. 2D contradict to results in Fig. 2A,B (all based on simulated data): In Fig. 2D, accuracy of a software tool (e.g., Conbase) decreases linearly when pDO increases from 0 to 0.9, but in Fig. 2A,B, sensitivity and specificity of Conbase (other tools as well) are almost always close to 1 when pDO is less than 0.5.

3. Fig. 1B already indicates that sensitivity of Conbase and LiRA cannot be more than 50%, because the other parts of genome cannot be analyzed by the two software tools while Monovar and SCcaller can analyze ~85% of the genome. However, when evaluating sensitivity in Fig. 2A, the authors neglected that Conbase and LiRA missed 50% of the genome while other tools may only miss 15% of the genome.

4. Heterozygous to homozygous ratio in simulated dataset for Fig. 2A is 1:1. However, this is an arbitrary ratio, because the chance that a same somatic mutation occurs at both alleles of a diploid genome is next to 0. Assuming the frequency of heterozygous mutations is 10E-7 per bp in normal somatic cells and 10E-5 per bp in tumor cells, the frequency of homozygous mutations is 10E-7*10E-7 and 10E-5*10E-5 in normal and tumor cells separately. Therefore, the heterozygous to homozygous ratio should be between 10E7:1 and 10E5:1.

5. The usage of EAL, i.e., errors from alignment artifact, is strange. EAL was introduced in line 262 as a major source of errors affecting variant calling in single cell sequencing. However, it is not clear what EAL exactly means (is it mapQ?). The authors tested the effect by EAL in their simulated data (Fig. 2C), but authors should provide evidence that EAL exists (and/or distribution of probabilities of EAL) in real publicly available dataset.

Other comments,

a. From a same dataset, results in Fig. 3 left panel suggest that there are potentially thousands of biologically plausible mutations in the dataset, but it is strange to see that Conbase only reported ~ 10 (Fig. 3 right panel). The same applies to Fig. 5 A and B.

b. In the response to my previous comment #1, the authors mentioned that Conbase can detect mutations at low read depth (down to 2x). However, because the level of error rate of NGS base calling, it is typical to use a minimum read depth of 4 (e.g., VarScan) or 3 even for bulk sequencing. The authors should provide evidence to support their claim.

**Authors Response**

**Point-by-point responses to the reviewers’ comments:**

Reviewer #1: The manuscript has been extensively revised. I have no further comments.

*Response:*

*We are happy that this reviewer acknowledges our efforts to extensively revise our original manuscript in response to the suggestions we received from Reviewer 1 and Reviewer 2.*

Reviewer #2: In the revised manuscript, the authors included the comparison with LiRA and SCcaller, and changed the strategy of generating simulated dataset for testing purpose.

*Response:*

*It is correct that we included comparative analyses with LiRA and SCcaller in our revised manuscript, as requested by this reviewer. Based on the suggestions from Reviewer 1, we updated our simulation strategy, to better model the complexity of the human genome.*

However, as I pointed out previously, simulated dataset may be problematic, and reviewer 1 was in agreement with me and suggested that simulated dataset may oversimplify real situation. This problem can be solved by taking my suggestion to use real dataset publicly available (e.g., Lodato etal Science 2015, Dong et al Nat Methods 2017, Chen et al Science 2017). However, the authors disagreed and did not perform testing using public dataset. Below, I list the evidence that the current simulation is still problematic or misleading, which are the reasons that testing on real datasets publicly available (accepted) are necessary.

*Response:*

*In accordance with instructions received from the editor, we have not included analysis of additional real datasets in our rerevised manuscript, on the basis that the simulations and real data presented in our revised manuscript are sufficient.*

*Please see our responses below, where we address all concerns of this reviewer and how these points are clarified in our rerevised manuscript.*

1. Sensitivities of SCcaller and Monovar estimated using the simulated dataset (Fig. 2A) contradicts to those estimated using real dataset (fig. 2c from Dong X et al. Nat Methods 2017).

*Response:*

*Figure 2c from Dong et al shows detection of germline SNVs in single cells, and not sensitivity. The purpose of our tool is to identify somatic variation in single cells, and therefore, germline SNVs are classified as Negatives in our analyses.*

*The sensitivity of SCcaller is instead shown in Figure 2a-b from Dong et al, and it is correct that the sensitivity determined by Dong et al is higher than the sensitivity shown in Figure 2a in our revised manuscript. However, SCcaller is expected to perform worse on data with uneven read coverage (confirmed by personal communication with the authors), as is the case for the results shown in Figure 2a in our revised manuscript and our rerevised manuscript. For this reason, we performed a second simulation with even coverage, representing data with uniform amplification. As expected, we find that the sensitivity for SCcaller is substantially improved, shown in Supplementary Figure 3 in our revised manuscript and rerevised manuscript. Comparing the results obtained from the second simulation with even coverage with the findings presented by Dong et al, we conclude that the estimated sensitivities correspond well in both studies. In our rerevised manuscript, we clarify that the sensitivity of SCcaller, tested in Figure 2a, (uneven coverage) and make more explicit that the sensitivity is high for SCcaller in data with even coverage as shown in Supplementary Figure 3b.*

*Furthermore, we note that the false discovery rate determined for Monovar in Figure 2d-e from Dong et al corresponds well with the high false discovery rate observed for Monovar in our analyses of our real datasets (Figure 3, Figure 4b, Figure 5b-d, Supplementary Figure 8 in our rerevised manuscript). In our rerevised manuscript, we discuss this observation.*

2. Results in Fig. 2D contradict to results in Fig. 2A,B (all based on simulated data): In Fig. 2D, accuracy of a software tool (e.g., Conbase) decreases linearly when pDO increases from 0 to 0.9, but in Fig. 2A,B, sensitivity and specificity of Conbase (other tools as well) are almost always close to 1 when pDO is less than 0.5.

*Response:*

*There is no conflict between the results shown in Figure 2a-b and 2d, which are two separate experiments. Figure 2a-b measures how well the methods detect somatic variant sites in a population of cells at the presence of artifacts. Figure 2d measures how well the methods predict genotypes in individual cells in somatic variant sites.*

*We apologize that this was not more clearly explained in our revised manuscript. In our rerevised manuscript, we explain that we perform two simulation experiments to test different prediction targets on lines 199-202 of the revised manuscript. On line 202-204 we explain that the first experiment tests how well the methods detect somatic variant sites in a population of cells. On line 232-233 we explain that the second experiment tests how well the methods predict genotypes in individual single cells in somatic variant sites.*

*To more clearly highlight the differences between these two simulation experiments, we have rewritten the figure legend to Figure 2 and Supplementary Figure in our rerevised manuscript.*

3. Fig. 1B already indicates that sensitivity of Conbase and LiRA cannot be more than 50%, because the other parts of genome cannot be analyzed by the two software tools while Monovar and SCcaller can analyze ~85% of the genome. However, when evaluating sensitivity in Fig. 2A, the authors neglected that Conbase and LiRA missed 50% of the genome while other tools may only miss 15% of the genome.

*Response:*

*We aimed to be very open about the fact that read phasing comes with the cost of lower sensitivity. In our revised manuscript, this was done by adding Figure 1b as well as discussing this issue in multiple sections. However, we agree with this reviewer that we could be even more clear about this fact. To present our results in a more balanced way, we have updated the introduction and extensively rewritten the discussion in our rerevised manuscript. We now state that there is a tradeoff between false discovery rate and sensitivity, where methods that integrate read phasing (Conbase and LiRA) achieve low false discovery rates whereas methods that do not integrate read phasing (Monovar and SCcaller) achieve better sensitivity. A user’s choice of method will depend on the downstream application, where Conbase is a best choice when high quality variant calls are more important than the sheer number of calls, for example in phylogenetic analysis or high resolution lineage tracing at the single cell level. In our rerevised manuscript, we state that Conbase may not be the optimal choice for other applications requiring a large total number of variant calls regardless of the abundance of false calls.*

4. Heterozygous to homozygous ratio in simulated dataset for Fig. 2A is 1:1. However, this is an arbitrary ratio, because the chance that a same somatic mutation occurs at both alleles of a diploid genome is next to 0. Assuming the frequency of heterozygous mutations is 10E-7 per bp in normal somatic cells and 10E-5 per bp in tumor cells, the frequency of homozygous mutations is 10E-7*10E-7 and 10E-5*10E-5 in normal and tumor cells separately. Therefore, the heterozygous to homozygous ratio should be between 10E7:1 and 10E5:1.

*Response:*

*In our simulations, we do not include homozygous mutations. The homozygous genotypes in our simulations represent samples with unmutated genotypes which are homozygous for the reference allele. The reason for not simulating homozygous somatic mutations is because such events would be extremely rare, as pointed out by this reviewer. In our rerevised manuscript, we state that homozygous somatic mutations are expected to be extremely rare in single cells (lines 209-211, 237-239).*

*On lines 207-209, 211-213, 239-242 in our rerevised manuscript, we clarify that homozygous genotypes in our simulations represent the ancestral, unmutated genotype state which is homozygous for the reference allele. We have also updated the color key and figure legend of Figure 2 and Supplementary Figure 3 in order to more clearly explain that the homozygous genotypes in our simulations represent the ancestral, unmutated state.*

5. The usage of EAL, i.e., errors from alignment artifact, is strange. EAL was introduced in line 262 as a major source of errors affecting variant calling in single cell sequencing. However, it is not clear what EAL exactly means (is it mapQ?). The authors tested the effect by EAL in their simulated data (Fig. 2C), but authors should provide evidence that EAL exists (and/or distribution of probabilities of EAL) in real publicly available dataset.

*Response:*

*It is well established that alignment artifacts are a major source of false positive variant calls (Li Bioinformatics 2014, Zafar et al Nature Methods 2016, Ju et al Nature 2017, Shi et al Cell Reports 2018, Lee-Six et al Nature 2018). As stated in our rerevised manuscript, EAL (abbreviation for error from ambiguous alignment) is an event that results in ambiguous read mapping in genomic regions affected by copy number variation and high sequence homology, including low-complexity genomic DNA (Li Bioinformatics 2014, Zafar et al Nature Methods 2016, Ju et al Nature 2017, Shi et al Cell Reports 2018, Lee-Six et al Nature 2018). The conventional approach is to exclude variants called in regions where alignment artifacts occur (Li Bioinformatics 2014, Zafar et al Nature Methods 2016, Ju et al Nature 2017, Shi et al Cell Reports 2018, Lee-Six et al Nature 2018). This is typically done by filtering sites where the observed read depth in a bulk sample exceeds the expected average read depth, and/or by excluding variant sites in close proximity to another variant site (Li Bioinformatics 2014, Zafar et al Nature Methods 2016, Ju et al Nature 2017, Shi et al Cell Reports 2018, Lee-Six et al Nature 2018). The maximal read depth filter is however not effective on single cell data due to amplification bias, and filtering variant sites based on proximity to other called variants has limited effect on single cell data due to allelic dropout. However, read phasing enables detection of discordant alleles which are indicative of the presence of signals derived from more than two haplotypes in mapped read data (Lodato et al Science 2018). Thus, integration of read phasing during variant calling provides a powerful tool to reduce the number of false variants called in regions affected by copy number variation and ambiguous read mapping in single cells.*

*MapQ is a measurement of how well a read is mapped to the reference genome. Because EAL is an event and MapQ is a metric, these two concepts are fundamentally different. In theory, the MapQ measurement could be affected by EAL events, provided that sufficient information is present in the reference genome. In practice, however, reads can still obtain a high MapQ score despite being present in segmental duplications that is absent in the reference genome. This phenomenon is well described by Heng Li (Bioinformatics 2014) where the effect of ambiguous read mapping on variant calling was evaluated for the two most widely used short read aligners for whole genome sequencing data, including Bowtie2 (Langmead and Salzberg Nature Methods 2012) and Burrows-Wheeler Alignment tool (BWA) (Li and Durbin Bioinformatics 2012). Li (Bioinformatics 2014) conclude that the two major sources of false positives in variant calling arise from alignment artifacts in low complexity regions and the limitation of using a reference genome with respect to the genome of the donor. EAL was introduced in our manuscript citing several references discussing this issue, which in turn provide evidence. We merely formalized the description of the problem, in order to generate a simulation model that is biologically relevant while taking the technical limitations upstream of variant calling into account.*

Other comments

a. From a same dataset, results in Fig. 3 left panel suggest that there are potentially thousands of biologically plausible mutations in the dataset, but it is strange to see that Conbase only reported ~ 10 (Fig. 3 right panel). The same applies to Fig. 5 A and B.

*Response:*

*This reviewer is correct in that the raw variant calling output from Monovar include a higher number of sites with plausible genotype distributions as compared to Conbase (Figure 3 left and right panel, Figure 5a-b). However, when applying thresholds for genotype quality on Monovar output, fewer plausible sites passed filters as compared to the number of sites detected by Conbase (Figure 3 middle and right panel, Figure 5a, c-d).*

*While Monovar detects more sites when no thresholds for genotype quality are applied, this comes with the cost of a substantially higher false discovery rate and much lower specificity. Conbase detects fewer sites, but achieves a low false discovery rate. In our rerevised manuscript, we have extensively rewritten our introduction and discussion, in order to present our results in a more balanced way. We conclude that the optimal choice of method for single cell variant calling, depend on what downstream application the predicted genotypes will be used for. Given the low false discovery rate, Conbase is the best choice for obtaining genotype data for lineage reconstruction and phylogenetic analysis, but may not be the best choice for other types of applications that require a large total number of variant calls regardless of the abundance of false variant calls. This has been clarified in our rerevised manuscript.*

b. In the response to my previous comment #1, the authors mentioned that Conbase can detect mutations at low read depth (down to 2x). However, because the level of error rate of NGS base calling, it is typical to use a minimum read depth of 4 (e.g., VarScan) or 3 even for bulk sequencing. The authors should provide evidence to support their claim.

*Response:*

*The ability of Conbase to detect true clonal somatic mutations at low read depth (2x) is evident from our analyses of real data where the read depth threshold was set to 2x (Figure 4a, left panel).*

*The strength of Conbase is that it considers compatibility among cells from the same individual and at the same time uses read phasing. This makes Conbase less sensitive to PCR and base calling errors since they are unlikely to occur on the same allele in multiple cells.*
